# Engineering the GH1 β-glucosidase from *Humicola insolens*: Insights on the stimulation of activity by glucose and xylose

**DOI:** 10.1371/journal.pone.0188254

**Published:** 2017-11-16

**Authors:** Luana Parras Meleiro, José Carlos Santos Salgado, Raquel Fonseca Maldonado, Sibeli Carli, Luiz Alberto Beraldo Moraes, Richard John Ward, João Atílio Jorge, Rosa Prazeres Melo Furriel

**Affiliations:** 1 Departamento de Química, Faculdade de Filosofia, Ciências e Letras de Ribeirão Preto, Universidade de São Paulo, Ribeirão Preto, São Paulo, Brasil; 2 Departamento de Bioquímica e Imunologia, Faculdade de Medicina de Ribeirão Preto, Universidade de São Paulo, Ribeirão Preto, São Paulo, Brasil; 3 Instituto Federal de Educação, Ciência e Tecnologia de São Paulo, São José dos Campos, São Paulo, Brasil; 4 Departamento de Biologia, Faculdade de Filosofia, Ciências e Letras de Ribeirão Preto, Universidade de São Paulo, Ribeirão Preto, São Paulo, Brasil; Kyung Hee Univeristy, REPUBLIC OF KOREA

## Abstract

The activity of the GH1 β-glucosidase from *Humicola insolens* (Bglhi) against *p*-nitrophenyl-β-D-glucopyranoside (*p*NP-Glc) and cellobiose is enhanced 2-fold by glucose and/or xylose. Kinetic and transglycosylation data showed that hydrolysis is preferred in the absence of monosaccharides. Stimulation involves allosteric interactions, increased transglycosylation and competition of the substrate and monosaccharides for the -1 glycone and the +1/+2 aglycone binding sites. Protein directed evolution has been used to generate 6 mutants of Bglhi with altered stimulation patterns. All mutants contain one of three substitutions (N235S, D237V or H307Y) clustered around the +1/+2 aglycone binding sites. Two mutants with the H307Y substitution preferentially followed the transglycosylation route in the absence of xylose or glucose. The strong stimulation of their *p*NP-glucosidase and cellobiase activities was accompanied by increased transglycosylation and higher monosaccharide tolerance. The D237V mutation favoured hydrolysis over transglycosylation and the *p*NP-glucosidase activity, but not the cellobiase activity, was stimulated by xylose. The substitution N235S abolished the preference for hydrolysis or transglycosylation; the cellobiase, but not the *p*NP-glucosidase activity of the mutants was strongly inhibited by xylose. Both the D237V and N235S mutations lowered tolerance to the monosaccharides. These results provide evidence that the fine modulation of the activity of Bglhi and mutants by glucose and/or xylose is regulated by the relative affinities of the glycone and aglycone binding sites for the substrate and the free monosaccharides.

## Introduction

β-glucosidases (β-D-glucoside glucohydrolases [E.C.3.2.1.21]) catalyze the hydrolysis of alkyl- and aryl-β-D-glucosides, cyanogenic glucosides, disaccharides and short oligosaccharides. Found in all domains of living organisms, they are represented in glycosyl hydrolase (GH) families GH1, GH2, GH3, GH5, GH9, GH30, GH39 and GH116 [1–4].

The β-glucosidases have attracted considerable attention due to their potential application in biotechnological processes, particularly the production of bioethanol from lignocellulosic biomass, where they are considered key enzymes for the complete hydrolysis of cellulose [4]. The β-glucosidases play a dual role by catalyzing the hydrolysis of cellobiose and soluble cellodextrins to glucose, and by reducing the reaction product inhibition of endo- and exoglucanases [5]. A major limitation for their application in saccharification of lignocellulosic biomass is that many known β-glucosidases are strongly inhibited by glucose, with K_i_ values in the low millimolar range [1,5–7].

In the current consensus, the enzymatic hydrolysis of cellulose for second generation ethanol production would be performed using high initial solid loading in order to reduce water usage and costs. This results in accumulation of polysaccharide hydrolysis products, where the concentration of glucose may reach hundreds of millimolar [5,8,9]. There is therefore a growing interest in enzymes that are glucose tolerant, and recently β-glucosidases tolerant to, or even stimulated by glucose have been described [5, 9–28]. However, the majority of these studies have been conducted with the exclusive use of the synthetic substrate *p*-nitrophenyl-β-D-glucopyranoside (*p*NP-Glc), where the tolerance/stimulation by glucose was investigated not by following the liberation of free glucose, but by release of *p*-nitrophenolate [9,12,16–18, 20–28].

**Fig 1:**
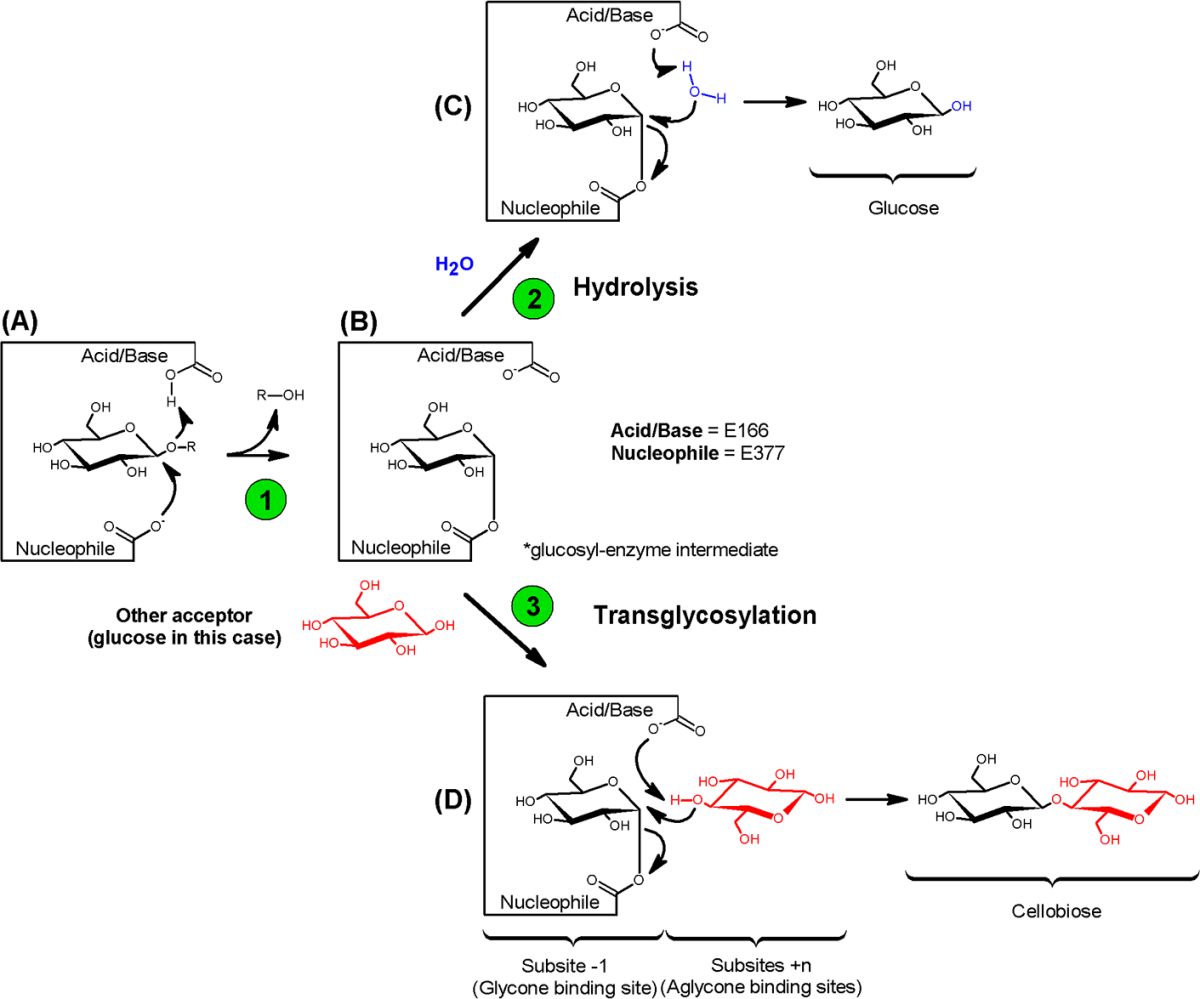
Catalytic reaction mechanism of the retaining β-glycosidases. After the formation of the glucosyl-enzyme intermediate (step 1), the entry of a water molecule leads to hydrolysis (step 2) and the entry of a sugar leads to transglycosylation (step 3).

Most known glucose tolerant or stimulated enzymes belong to the GH1 family. In the retaining catalytic mechanism (Fig 1), one of the two glutamate residues in the GH1 β-glucosidase active site (Fig 1A) acts as an acid/base catalyst and the other acts as a nucleophile [2,29]. The cleavage of the glycosidic bond occurs in two steps, denominated as glycosylation and deglycosylation, respectively. Glycosylation involves the formation (Fig 1, step 1) of a covalent glucosyl-enzyme intermediate (Fig 1B), which is subsequently deglycosylated in a hydrolysis reaction (Fig 1, step 2) through the attack of a water molecule, thereby liberating glucose and restoring the free enzyme (Fig 1C). Under certain conditions, however, the attack on the glucosyl-enzyme intermediate may be carried out not by water, but by another acceptor nucleophile such as a monosaccharide, disaccharide, aryl-, alkyl-alcohol or monoterpene alcohol (Fig 1D), resulting in a transglycosylation reaction (Fig 1, step 3) [30,31].

Although the catalytic mechanism of the GH1 β-glucosidases has been confirmed experimentally [32,33] only recently have the concurrent reactions of hydrolysis and transglycosylation been recognized as being crucial for the understanding of the glucose stimulation of the GH1 β-glucosidases [19,26,27], a property which had previously been attributed to allosteric effects [5, 13–15,34] or to a reduction in substrate inhibition [28]. The topology of the active site has also been associated with the tolerance of some enzymes to high concentration of glucose [28,29,35]. Furthermore, a recent theoretical study raised the possibility that competition of inhibitors with the non-productive binding of substrate may be a possible mechanism of glucose/xylose stimulation of β-glucosidases that have more than one glycosyl residue binding subsite in the aglycone binding site [36]. It has been recently proposed that the relative binding affinity/preference by sites at the entrance and middle of the substrate channel regulate the effects of glucose [37].

Filamentous fungi of the genus *Humicola* are excellent producers of various cellulolytic enzymes, including several β-glucosidases [38]. To date, an intracellular GH1 β-glucosidase (Bglhi) [14], an extracellular GH3 β-glucosidase (BglHi2) [39] and three heterologous GH3 β-glucosidase isoenzymes from *Humicola insolens* expressed in *Pichia pastoris* (HiBgl3A, HiBgl3B and HiBgl3C) [40] have been characterized. With the exception of the BglHi2, these enzymes present high glucose tolerance [14,39,40]. We have recently described the catalytic properties of the Bglhi, which in addition to high glucose and thermal tolerance was also shown to have high catalytic efficiency for cellobiose hydrolysis. Furthermore, catalytic activity against both *p*NP-Glc and cellobiose was stimulated 2-fold by glucose and/or xylose [14,34], suggesting that this β-glucosidase is therefore an interesting candidate for application in industrial processes for the hydrolysis of lignocellulosic materials. The recombinant Bglhi enzyme expressed in *Escherichia coli* maintained both an elevated catalytic efficiency for cellobiose hydrolysis and glucose and xylose tolerance/stimulation [15]. In addition, the three-dimensional structure of the Bglhi has been solved [29], and the enzyme has been useful to study the mechanism of stimulation of GH1 β-glucosidases by the reaction product.

In the present study, a directed evolution approach using error prone PCR has been applied to create a series of Bglhi mutants with altered catalytic properties with respect to glucose/xylose stimulation and/or tolerance. The characterization of these mutants, particularly with respect to their action on the natural substrate cellobiose, has contributed to elucidate the structural determinants and the dynamics of the phenomenon of glucose and xylose stimulation of this GH1 β-glucosidase.

## Materials and methods

### Construction of a random mutant library

The construction of the pET28_*bglhi* plasmid containing the Bglhi has been previously described [15], and random mutagenesis of the *bglhi* coding sequence was performed by error-prone PCR (epPCR) using the pET28_*bglhi* plasmid as template. The reaction mixture (100 μL) contained 7 mM MgCl_2_, 200 μM dNTPs, 100 pmol of each universal DNA primers (T7promoter and T7terminator, complementary to the 5´ and 3´ regions flanking the *bglhi* coding sequence in the pET-28a(+) vector), 200 ng template, 100 μM MnCl_2_ and 5 units (U) of Taq polymerase (Thermo Scientific, Wilmington, USA) and its respective reaction buffer. Thermocyling involved a single initial heating to 95°C for 2 min, followed by 30 cycles of 94°C for 1 min, 45°C for 1 min and 72°C for 3 min and a final heating to 72°C for 10 min. The PCR products were separated by agarose gel electrophoresis and purified by Wizard® SV Gel Extraction kit (Promega, Madison, USA). The extracted DNA fragments were digested with restriction enzymes *Nhe*I and *Bam*HI (Thermo Scientific, Waltham, MA, USA), and ligated into the pET-28a(+) vector (Novagen, Madison, WI, USA) linearized with the same enzymes. The random mutant library (denominated pET28_mut) was used to transform *E*. *coli* BL21 (DE3) by electroporation. The recombinant plasmid pET28_*bglhi* was also used to transform *E*. *coli* BL21 (DE3) as a control for all random mutant library screening steps.

### Random mutant library screening

The *E*. *coli* cells transformed with either the pET28_mut library, or the pET28_*bglhi* as positive control, were plated on Luria-Bertaini broth (LB)-agar plates containing 40 μg mL^-1^ kanamycin and 34 μg mL^−1^ chloramphenicol. After incubation at 37°C for approximately 16 h, individual colonies were transferred to 96-well microplates containing 200 μL LB medium supplemented with the same antibiotics using an automated colony picker (model K6, Kbiosystems, Basildon, Essex, UK). The 96-well plates were incubated at 37°C for 16 h, and replicated onto 250 x 250 mm bioassay plates containing LB-agar including the same antibiotics, 50 μmol L^-1^ isopropyl-β-D-1-thiogalactopyranoside (IPTG), 1 g L^-1^ esculin and 0.3 g L^-1^ ferric chloride. After incubation of the plates at 37°C for 16 h, the clones expressing β-glucosidase activity were identified by the formation of dark halos surrounding the colonies. These colonies were manually transferred to two separate 96-well microplates containing 200 μL LB-medium, specific antibiotics and IPTG at the same concentrations. After incubation of the plates at 37°C for 16 h, one of the plates was supplemented with 20% (v/v) glycerol and stored at -80°C. The other plate was centrifuged at 4000 x *g* for 15 min, and the supernatants were used for enzymatic assays in 96-well plates at 50°C. After the extended time of expression, spontaneous cell lysis releases proteins that can be detected in the culture supernatant. After adequate dilution, ten-microliter aliquots of each culture supernatant were mixed with 80 μL of a reaction medium containing 2 mM *p*NP-Glc in 50 mM Bis-Tris buffer, pH 6.0, in the presence or absence of 100 mM glucose or xylose. After 5 min the reactions were interrupted by the addition of 160 μL of a saturated sodium tetraborate solution and the *p*NP-glucosidase activities were determined (see the “Enzymatic assays” section). For each selected clone, two stimulation factors (SF) were calculated from the measured activities: SF_Glc_, which was defined as the ratio of the activity in the presence of 100 mM glucose to the activity without glucose; and SF_Xyl_, which was defined as the activity at 100 mM xylose to the activity without xylose. The wild type Bglhi (SF_Glc_ = 1.8; SF_Xyl_ = 2.0) was employed as a control throughout the screening procedure, and those clones with an SF lower than 1.3 or greater than 2.2 were selected and the mutations were identified by nucleotide sequencing of the plasmid DNA. Each enzymatic assay was performed in duplicate and the experiments were repeated three times with each monosaccharide (glucose and xylose). The SF values presented in the accompanying tables are the mean ± SD for these triplicate measurements (n = 3).

### Site-directed mutagenesis

The single mutation H307Y was generated by PCR based site-directed mutagenesis [41], where the PCR reaction contained 10 ng of plasmid pET28_*bglhi* as template, 50 pmol of the primers 5’-GGCATGAACTACTACACGGCCAACTACATCAAGCAC-3’ (forward) and 5’- CGTGTAGTAGTTCATGCCGTAGAAGTCGTTGGAGCC-3’ (reverse), 200 μM dNTPs and 2.5 U of *Pfu* DNA polymerase (Thermo Scientific, Waltham, MA, USA) and its respective buffer. The PCR was performed with initial denaturation of the template DNA at 95°C for 3 min, followed by 30 cycles of 95°C for 1 min, 50°C for 1 min, 72ºC for 15 min and a final extension step at 72°C for 10 min. After PCR, the product was incubated with 2 U of *Dpn*I (Thermo Scientific, Waltham, MA, USA) at 37°C for 3 h to digest the methylated template DNA. One microliter of the *Dpn*I digested sample was used to transform *E*. *coli* DH5α. After nucleotide sequencing confirm the mutation, the plasmid (denominated pET28_307) was subsequently used to transform *E*. *coli* BL21 (DE3) for the expression of the recombinant enzyme (See the “Protein expression and purification” section).

### Protein expression and purification

The Bglhi and the selected mutants were overexpressed in *E*. *coli* BL21 (DE3) after transformation with the plasmids pET-28a(+) containing the respective coding sequences. The cells were grown in HDM medium (25 g L^−1^ yeast extract, 15 g L^−1^ tryptone and 10 mM MgSO_4_) supplemented with specific antibiotics at 37°C to an OD_600_ of approximately 0.6. Protein expression was induced by adding IPTG to the culture media at a final concentration of 1 mM. After 5 h induction at 37°C, the cells were collected by centrifugation at 5000 x *g* for 20 min at 4°C. The pellets were resuspended in lysis buffer (50 mM HEPES buffer, pH 8.0, containing 500 mM NaCl and 1% (v/v) Triton X-100) and disrupted by sonication. Cell debris were removed by centrifugation and the recombinant proteins were purified from the supernatant using nickel affinity chromatography (HisLink™, Promega), according to the manufacturer’s instructions. The protein concentrations were determined according to Read and Northcote [42], using bovine serum albumin as the standard.

### Electrophoresis

Electrophoresis under denaturing conditions (SDS-PAGE) was performed in 10% acrylamide slab gels, according to Laemmli [43], using a pre-stained molecular weight marker (6.1–204.3 kDa, Sigma-Aldrich Chem. Co., St Louis, MO, USA). The DNA analyses were performed in 1% (w/v) agarose gels (Agargen, Madrid, Spain) containing 0.02% SYBR Safe DNA Gel Stain (Invitrogen, Carlsbad, CA, USA), and the bands were visualized under UV light.

### Enzymatic assays

The *p*NP-glucosidase activity of Bglhi and mutants was defined as the rate of liberation of *p*-nitrophenolate ion (*p*NP^-^) and was assayed spectrophotometrically as previously described [14]. The first reaction step (formation of the glucosyl-enzyme intermediate, Fig 1) releases *p-*Nitrophenol, and is a product that is common to both the hydrolysis and the transglycosylation routes. Unless otherwise stated, the *p*NP-glucosidase activity was determined at 90–95% saturating substrate concentrations, which were determined for each enzyme (2 mM for Bglhi, N89Y/H307Y and H307Y; 1.5 mM N235S, A141T/N235S and D237V/P389H/E395G/K475R, and 3 mM for D237V). Standard assays conditions were 50°C and 50 mM Bis-Tris buffer, pH 6.0, for Bglhi, N89Y/H307Y and H307Y; 50°C and McIlvaine buffer [44], pH 5.5 or 5.0, for the N235S and A141T/N235S mutants, respectively; 45°C and 50 mM sodium acetate buffer, pH 5.5 or 5.0, for D237V/P389H/E395G/K475R and D237V, respectively.

The cellobiase activity was defined as the rate of free glucose liberation with cellobiose as substrate, where a free glucose molecule is the first product released in the glycosylation step of the catalytic cycle (Fig 1 step 1), and an additional free glucose is generated by the hydrolysis of the glycosyl-enzyme adduct (Fig 1 step 2). When transglycosylation occurs (Fig 1 step 3) oligosaccharides are generated, resulting in reduced free glucose:cellobiose ratios and the accumulation of cello-oligosacchrides. Unless otherwise stated, the cellobiase activity was assayed under the same pH and temperature conditions as described for the *p*NP-glucosidase activity and at 90–95% saturating cellobiose substrate conditions (5 mM for Bglhi; 40 mM for N89Y/H307Y, 70 mM for H307Y and D237V; 1 mM for N235S; 2 mM for A141T/N235S and 10 mM for D237V/P389H/E395G/K475R). The reactions were interrupted by boiling for 10 min and the free glucose concentration was quantified using the glucose assay kit GAHK20 (Sigma-Aldrich Chem. Co., St Louis, MO, USA) according to the manufacturer’s instructions.

The experimental conditions (reaction times, enzymatic units) for the activity measurements were adjusted for each mutant to guarantee the estimation of initial velocities (linear response of product formation with the reaction time). One enzyme unit (U) was defined as the amount of enzyme that releases 1 μmol of product per min. Specific activity was defined as U per milligram total protein (U mg^-1^).

### Determination of kinetic parameters

The kinetic parameters for the release of *p*NP^-^ from *p*NP-Glc and the release of free glucose from cellobiose by Bglhi and mutants were calculated using the SigrafW Software which fits experimental data to the Hill equation using non-linear regression [45]. For all enzymes, values were estimated for the V_max_ (maximum velocity), K_M*p*NP-Glc_ (enzyme-substrate apparent dissociation constant with the *p*NP-Glc substrate) K_Mcellobiose_ (enzyme-substrate apparent dissociation constant with the cellobiose substrate), K_aGlc_ (the glucose concentration needed for half-maximal activation of the enzyme), K_aXyl_ (the xylose concentration needed for half-maximal activation of the enzyme) and n_H_ (the Hill coefficient). The *k*_cat_ was defined as the number of molecules of *p*NP^-^ or free glucose liberated from pNP-Glc or cellobiose, respectively, by one molecule of enzyme per second. The IC_50_ was defined as the concentration of glucose or xylose that inhibited 50% of the control activity determined at 90–95% saturating substrate concentration and in the absence of glucose or xylose, and was determined graphically from plots of activity as a function of glucose or xylose concentration.

All experimental kinetic curves were repeated three times using different pure recombinant enzyme preparations, where each experimental point was assayed in duplicate. The kinetic parameters presented are calculated values and are given as the mean ± SD of the three replicates (n = 3). Data fitting and statistical analyses were carried out using the OriginPro 8 SRO software package (OriginLab Corp., Northampton, MA, USA).

### Determination of the ratios of *p*NP-Glc hydrolysis and transglycosylation

The determination of the rates of *p*NP^-^ and free glucose release from *p*NP-Glc allows the estimation of the probabilities of the hydrolysis and the transglycosylation reactions catalyzed by a given mutant enzyme under particular experimental conditions. These probabilities are expressed as the fractions of reactions resulting in hydrolysis (%H) or transglycosylation (%T) [30].

To determine the effect of xylose on the values of %H and %T, enzymatic reactions were performed (see the “Enzymatic assays” section) in the absence or presence of xylose at a concentration that resulted in maximal stimulation of the enzymatic activity (MC_max_), which corresponded to 100 mM for Bglhi, 150 mM for the N89Y/H307Y and H307Y mutants, 200 mM for the D237V mutant, and 80 mM for the N235S, A141T/N235S and D237V/P389H/E395G/K475R mutants. For each enzyme, total enzyme units and time intervals were adjusted to achieve approximately 5% substrate consumption by the end of the experimental reaction (in the absence of xylose). The reactions were interrupted by boiling for 10 min and the sample volume reduced from 600 μL to 100 μL using a vacuum concentrator (Concentrator Plus 5301, Eppendorf Inc., NY, USA) at 45°C. The concentration of *p*NP^-^ in each reaction medium was determined spectrophotometrically according to Souza et al. [14] while free glucose was quantified using the glucose assay kit GAHK20 (Sigma-Aldrich) according to the manufacturer’s instructions. The enzymatic assays were repeated six times and the %H and %T values are given as means ± SD of the six calculated values (n = 6).

### Analysis of the reaction products of Bglhi and mutants

The reaction products resulting from the action of the wild type Bglhi and mutants against cellobiose were analyzed by thin layer chromatography (TLC) and mass spectrometry (MS). The reactions were performed at 90–95% saturating cellobiose concentration in the absence of xylose and glucose. Subsequently reactions were performed under various conditions as follows: i) in the presence of xylose or glucose at MC_max_ (100 mM for Bglhi, 200 mM for the N89Y/H307Y mutant and 500 mM for H307Y); ii) in the presence of xylose or glucose at the maximal concentration that did not apparently inhibit the enzymatic activity (maximum tolerance, MT), corresponding to 30 mM for D237V/P389H/E395G/K475R, 300 mM for D237V, 50 mM for A141T/N235S and 30 mM for N235S. The reactions were performed as described in the “Enzymatic assays” section, except that the reaction buffer was 10 mM ammonium acetate, pH 5.5. The total enzyme units of all enzymes were adjusted to achieve 5% substrate consumption after 5 min on the basis of glucose liberation in the absence of xylose. The time course of the formation of hydrolysis and transglycosylation products was followed from 10 min to 24 h.

TLC analyses were carried out on silica gel plates (DC-Alufolien Kieselgel 60, Merck, Darmstadt, Germany) at room temperature using ethyl acetate/acetic acid/formic acid/water (9:3:1:4, v/v/v/v) as the mobile phase. Volumes of the samples were spotted on the TLC plates, along with glucose (G_1_), cellobiose (G_2_), cellotriose (G_3_), cellotetraose (G_4_), xylose (X_1_), xylobiose (X_2_), gentibiose (Ge) and an equimolar mixture of sophorose (S) and cellobiose (SG_2_) as standards. After two runs, the products were detected by spraying the plates with 0.4% (w/v) orcinol in sulfuric acid/ethyl alcohol (1:9 v/v) followed by heating at 150°C to reveal the individual spots.

MS analyses were performed on a Xevo® TQ-S instrument (Waters Corporation, Milford, MS, USA) with an electrospray ionization source operated in positive mode. Five μL aliquots of the reaction samples were introduced into the mass spectrometer by direct insertion in acetonitrile/formic acid 0.1% (50% v/v) at a flow rate of 0.1 mL min^-1^. The ESI interface conditions were as follows: capillary voltage 3.2 kV, cone voltage 40 V, Z-spray source temperature 150°C, desolvation gas (N_2_) temperature 250°C, desolvation gas flow 600 L h^-1^ (mass range from *m/z* 100 to 1200). The experiments of tandem mass spectrometry (MS/MS) on precursor ions of interest were performed by collision-induced dissociation (CID) using argon gas as the collision gas. The collision energy was over the range from 10 to 50 eV. Data acquisition and processing were performed using the MassLynx V4.1 (Waters Corporation).

## Results

### Screening of random mutant library

Approximately 4,400 bacterial colonies were screened for β-glucosidase activity, and 418 clones that expressed active enzymes were identified. Screening of these 418 clones identified mutants showing pronounced differences in the stimulation of the *p*NP-glucosidase activity by glucose and xylose as compared to the wild type Bglhi. Five mutants exhibiting SF_Glc_ and/or SF_Xyl_ <1.3 or >2.2 were selected (Table 1). Nucleotide sequence analysis of these 5 mutants showed a mutation rate of 1.7 ± 0.5 bp per kb with amino acid substitutions ranging from one to four per mutant.

**Table 1:** Characterization of the selected mutants.

Mutant	Mutation	Amino acid	Substitution	SF	
				Glucose	Xylose
N235S	704 A → G	235	Asn → Ser	1.16	1.36
	717 C → T	*	*		
A141T/N235S	421 G → A	141	Ala → Thr	1.12	1.05
	704 A → G	235	Asn → Ser		
D237V	710 A → T	237	Asp → Val	1.28	1.16
D237V/P389H/E395G/K475R	710 A → T	237	Asp → Val	1.17	1.16
	879 C → T	*	*		
	1166 C → A	389	Pro → His		
	1184 A → G	395	Glu → Gly		
	1424 A → G	475	Lys → Arg		
N89Y/H307Y	265 A → T	89	Asn → Tyr	2.51	2.71
	919 C → T	307	His → Tyr		

* Silent mutations

### Structural mapping, overexpression and purification of Bglhi and its mutants

**Fig 2:**
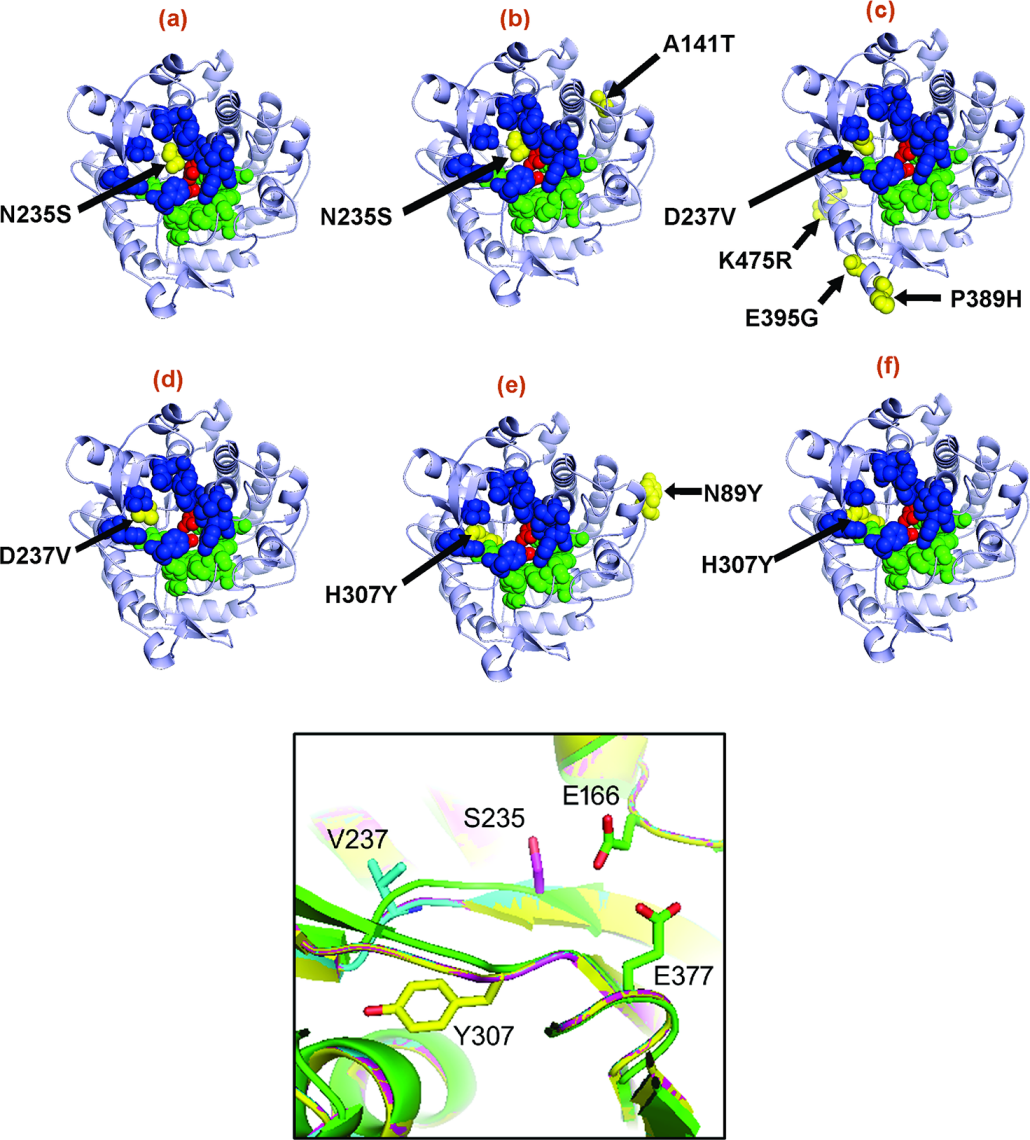
Positions of the mutations inserted in Bglhi in each mutant. The 3-D structures of (a) N235S, (b) A141T/N235S, (c) D237V/P389H/E395G/K475R, (d) D237V, (e) N89YH307Y and (f) H307Y were modelled using the crystal structure of Bglhi (PDB code: 4MDO) as template with the MODELLER 9.10 software. The amino acid substitutions identified by random mutagenesis and functional selection are indicated as yellow spheres in Fig 2. The catalytic residues (E166 and E377), the glycone and the aglycone-binding sites are shown as red, green and blue spheres, respectively. The inset shows a ribbon representation of the +1/+2 aglycone binding region of the wild-type Bglhi (in green) showing the catalytic residues (E166 and E377), together with the mutants N235S (in magenta), D237V (in light blue) and H307Y (in yellow). The highly conserved positions of the main chain atoms resulted in the mottled appearance of the main chain after superposition of the different structures.

The positions of the amino acid substitutions were mapped onto the Bglhi crystal structure (PDB 4MDO; [29]) (Fig 2). All selected mutants showed at least one mutation in the active site region of Bglhi, and additional peripheral substitutions occurred in mutants D237V/P389H/E395G/K475R, N89Y/H307Y and A141T/N235S. The mutant D237V showed a single substitution that was identical to that found in the active site region of D237V/P389H/E395G/K475R, while N235S showed the same substitution present in the active site region of A141T/N235S. The single mutant H307Y was constructed by site-directed mutagenesis to contain the same substitution present in the active site region of N89Y/H307Y. The wild type Bglhi, and the mutants N89Y/H307Y, H307Y, D237V/P389H/E395G/K475R, D237V, A141T/N235S and N235S were overexpressed in *E*. *coli* BL21 (DE3) as soluble proteins and purified to homogeneity (S1 Fig).

### Biochemical characterization and kinetic analysis of Bglhi and mutants using *p*NP-Glc as substrate

The *p*NP-glucosidase activity of Bglhi and mutants was analyzed between 30 to 65°C and over the pH range 4.0 to 8.0 (S2 Fig). The Bglhi activity gradually increased from 30 to 55°C, reaching a maximum at 60°C and abruptly decreasing at higher temperatures. Similar profiles were observed for the H307Y, A141T/N235S and N235S mutants, although with the maximum activity at 55°C. In contrast, alterations in the temperature/activity profiles were observed for the other mutants, with plateaus of maximum catalytic activity from 50–60°C, 40–50°C and 35–50°C for N89Y/H307Y, D237V/P389H/E395G/K475R and D237V, respectively. Similar profiles were observed for the pH optima for Bglhi, N89Y/H307Y and H307Y, with maximum activity plateaus from pH 5.0 to 7.0. However, catalytic activity profiles of as a function of pH were more restricted for D237V/P389H/E395G/K475R, D237V, A141T/N235S and N235S, with maximal activities at pH 5.5, 5.0, 5.0 and 5.5, respectively.

**Fig 3:**
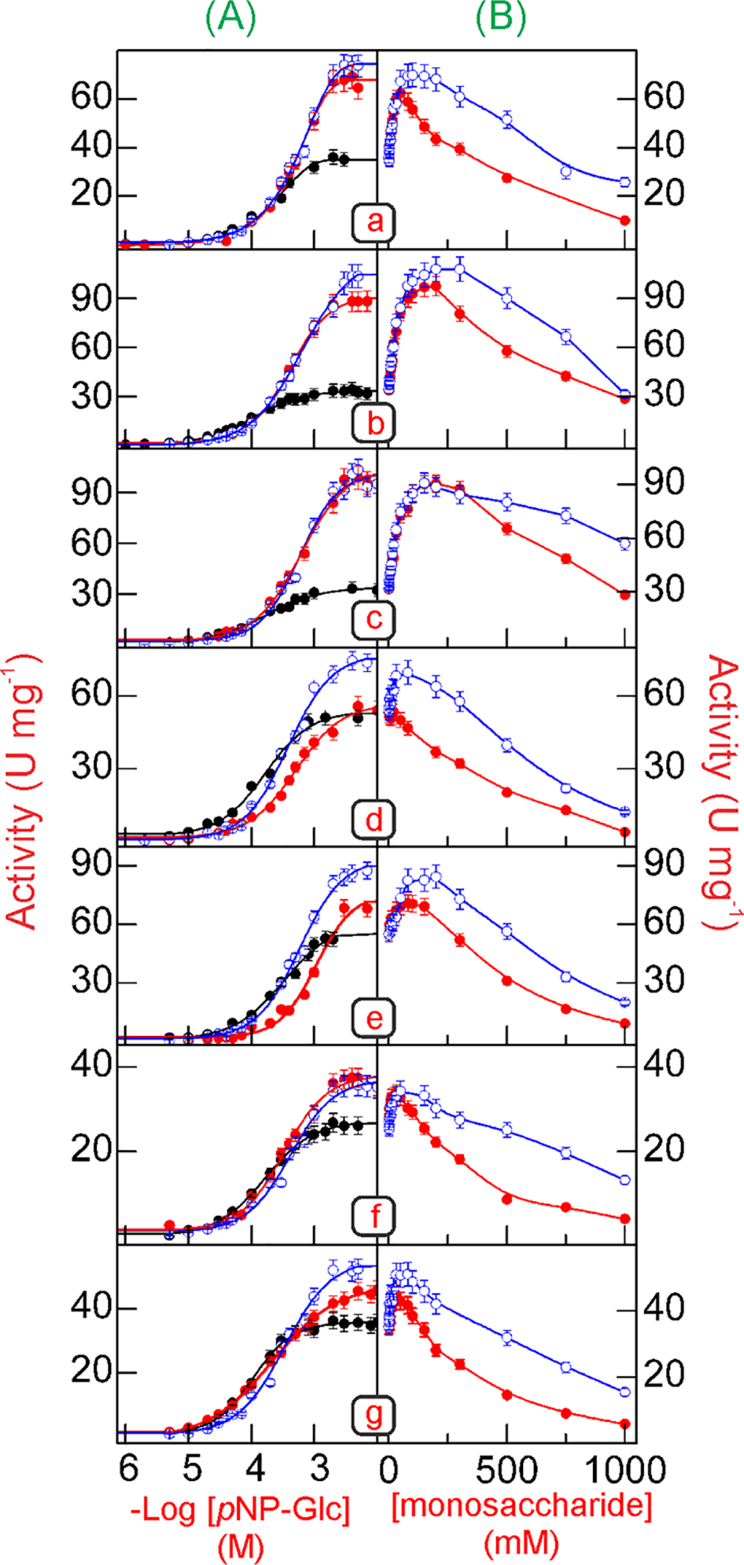
Effect of increasing concentrations of *p*NP-Glc, glucose and xylose on the *p*NP-glucosidase activity. (a) Bglhi, (b) N89Y/H307Y, (c) H307Y, (d) D237V/P389H/E395G/K475R, (e) D237V, (f) A141T/N235S, (g) N235S. **(A)** Effect of increasing concentrations of *p*NP-Glc on the *p*NP-glucosidase activity in the absence (black lines and dots) or in the presence of glucose (red lines and dots) or xylose (blue lines and dots) at fixed concentrations equal to MC_max_. **(B)** Effect of increasing concentrations of glucose (red lines and dots) or xylose (blue lines and dots) on the *p*NP-glucosidase activity of each mutant enzyme at 90–95% saturating concentrations of *p*NP-Glc (2 mM for Bglhi, N89Y/H307Y and H307Y; 1.5 mM for D237V/P389H/E395G/K475R, A141T/N235S and N235S; 3 mM for D237V). All experiments were repeated three times using three separate pure enzyme preparations. Each point represents the mean of duplicate assays ± SD (error bars are not evident, as they lie within the area of the symbol).

The kinetic parameters for the effect of *p*NP-Glc on the *p*NP-glucosidase activity of Bglhi and mutants in the absence or in the presence of xylose or glucose are presented in Fig 3A and Table 2. In the absence of monosaccharides, the stimulation of the wild type Bglhi and all mutants occurred as single curves following non-Michaelis-Menten kinetics (n_H_>1). Similar maximum velocities were observed for Bglhi, N89Y/H307Y, H307Y, A141T/N235S and N235S. In contrast, V_max_ values about 1.5-fold higher than the Bglhi were determined for D237V/P389H/E395G/K475R and D237V. With the exception of D237V, which showed a K_M*p*NP-Glc_ value about 1.3-fold higher, all other enzymes showed lower K_M*p*NP-Glc_ values, as compared to the wild type Bglhi. The efficiency of substrate utilization (*k*_cat_/K_M*p*NP-Glc_) determined for A141T/N235S was similar to that for the Bglhi. In contrast, catalytic efficiencies 1.8, 1.2-, 2.1-, 1.2- and 2.1-fold higher were estimated for N89Y/H307Y, H307Y, D237V/P389H/E395G/K475R, D237V and N235S, respectively.

**Table 2:** Kinetic parameters for the stimulation of the *p*NP-glucosidase activity of Bglhi and mutants against *p*NP-Glc in the absence or presence of fixed concentrations of glucose or xylose.

Enzyme	[Monosaccharide] (mM)	V_max_ (U mg^-1^)	K_M*p*NP-Glc_ (mM)	*k*_cat_/K_M*p*NP-Glc_ (s^-1^mM^-1^)	n_H_
Bglhi	0	37.3 ± 1.8	0.22 ± 0.01	158.53 ± 15.89	1.2
	Glucose (50)	72.1 ± 2.9	0.50 ± 0.02	134.83 ± 11.57	1.4
	Xylose (100)	77.3 ± 3.9	0.54 ± 0.03	133.84 ± 15.17	1.4
N89Y/H307Y	0	34.0 ± 1.7	0.11 ± 0.01	289.00 ± 43.55	1.1
	Glucose (100)	93.7 ± 3.7	0.43 ± 0.02	203.74 ± 18.74	1.1
	Xylose (150)	110.9 ± 3.3	0.57 ± 0.02	181.91 ± 12.62	1.1
H307Y	0	32.9 ± 1.6	0.16 ± 0.01	192.26 ± 22.85	1.1
	Glucose (100)	103.1 ± 6.1	0.56 ± 0.03	172.14 ± 20.76	1.2
	Xylose (150)	103.2 ± 7.2	0.57 ± 0.04	169.28 ± 25.34	1.3
D237V/P389H/E395G/K475R	0	54.7 ± 3.8	0.15 ± 0.01	340.96 ± 49.64	1.1
	Glucose (50)	58.3 ± 2.9	0.50 ± 0.03	109.02 ± 12.80	1.1
	Xylose (80)	77.7 ± 4.7	0.38 ± 0.02	191.18 ± 23.13	1.2
D237V	0	55.5 ± 3.3	0.28 ± 0.02	185.33 ± 25.94	1.2
	Glucose (100)	73.0 ± 5.1	0.95 ± 0.07	71.85 ± 11.03	1.4
	Xylose (200)	89.6 ± 6.3	0.52 ± 0.04	161.11 ± 25.37	1.3
A141T/N235S	0	27.1 ± 1.3	0.16 ± 0.01	158.37 ± 18.71	1.1
	Glucose (30)	38.7 ± 2.3	0.31 ± 0.02	116.72 ± 15.47	1.1
	Xylose (80)	37.6 ± 2.2	0.40 ± 0.02	87.89 ± 10.20	1.1
N235S	0	35.9 ± 2.9	0.10 ± 0.01	335.67 ± 64.90	1.3
	Glucose (30)	47.4 ± 3.8	0.22 ± 0.02	201.45 ± 36.86	1.3
	Xylose (80)	54.9 ± 3.8	0.32 ± 0.02	160.41 ± 22.60	1.1

Glucose and xylose concentrations (in brackets) are equal to the MC_max_ presented in Table 3. Each experimental kinetic curve was repeated three times, using three separate preparations of the pure enzyme. Each activity assay was performed in duplicate. The kinetic parameters are the means ± SD of the values calculated for each repetition (n = 3).

Fig 3B shows the effect of increasing glucose or xylose concentrations over the range 0–1000 mM on the *p*NP-glucosidase activity of wild type Bglhi and mutants at 90–95% saturating concentrations of substrate for each enzyme. In agreement with previous studies, the release of *p*NP^-^ by Bglhi was stimulated up to 1.9- and 2.1-fold by glucose and xylose, respectively, with MC_max_ values for glucose and xylose of 50 and 100 mM, respectively (Table 3). However, different profiles for the stimulation of *p*NP^-^ liberation by glucose or xylose were observed for the mutant enzymes. The *p*NP-glucosidase activity of the N89Y/H307Y and H307Y mutants was stimulated up to 2.8- and 3.1-fold by 100–200 mM glucose, and up to 3.3- and 3.1-fold by 150–200 mM xylose. In contrast, the other mutants presented lower SF values for both monosaccharides as compared to the wild type Bglhi. Remarkably, although the D237V/P389H/E395G/K475R mutant was not stimulated by glucose, this enzyme showed a SF_Xyl_ of about 1.4. Furthermore, the D237V/P389H/E395G/K475R, D237V, A141T/N235S and N235S mutants presented high variability of the MC_max_ for glucose and xylose (Table 3).

**Table 3:** Glucose- and xylose-stimulation of the *p*NP-glucosidase activity of Bglhi and mutants.

Enzyme	[*p*NP-Glc]	Monosaccharide	K_aGlc_ or K_aXyl_	SF	MC_max_	MT	n_H_
	(mM)		(mM)	(fold)	(mM)	(mM)	
Bglhi	2	Glucose	10.9 ± 0.5	1.9	50	~ 400	1.2
		Xylose	17.6 ± 1.1	2.1	100	~ 750	1.2
N89Y/H307Y	2	Glucose	28.5 ± 1,7	2.8	100–200	~ 1000	1.2
		Xylose	31.1 ± 2.5	3.3	150–200	~ 1000	1.4
H307Y	2	Glucose	42.1 ± 2.1	3.1	100–200	~ 1000	1.2
		Xylose	29.7 ± 1.6	3.1	150–200	> 1000	1.2
D237V/P389H/	1.5	Glucose	-	-	-	50	-
E395G/K475R		Xylose	16.7 ± 0.9	1.4	80	~ 340	1.1
D237V	3	Glucose	19.7 ± 1.1	1.3	80–100	300	1.9
		Xylose	36.2 ± 2.9	1.6	150–200	~ 500	1.4
A141T/N235S	1.5	Glucose	3.4 ± 0.2	1.4	30	150	1.2
		Xylose	8.7 ± 0.3	1.4	50–80	500	1.3
N235S	1.5	Glucose	5.8 ± 0.3	1.3	20–30	150	1.2
		Xylose	6.7 ± 0.1	1.5	30–80	500	1.1

Each experimental curve was repeated three times, using three separate preparations of the pure enzyme under 90–95% saturating substrate; each activity assay was performed in duplicate. The kinetic parameters (K_aGlc_, K_aXyl_ and n_H_) were determined at increasing concentrations of glucose or xylose within the stimulatory range (0-MC_max_), and are given as means ± SD of the values calculated for each repetition (n = 3).

In addition to the differences in the stimulatory effects, the mutant enzymes also showed variable tolerance to xylose and glucose, as reflected by their MT values (Table 3). While the N89Y/H307Y and H307Y mutants presented increased tolerance to both monosaccharides as compared to Bglhi, the other mutants showed significantly reduced tolerance, particularly to glucose. This corroborates with a quantitative analysis of glucose tolerance based on the IC_50_ values determined for each enzyme, which revealed values of 780 mM for Bglhi, and >1 M for N89Y/H307Y and H307Y, contrasting with 400, 580, 400 and 450 mM for D237V/P389H/E395G/K475R, D237V, A141T/N235S and N235S, respectively. For xylose, however, the IC_50_ values were >1 M for Bglhi, N89Y/H307Y and H307Y, and 700, 870, 1000 and 920 mM for D237V/P389H/E395G/K475R, D237V, A141T/N235S and N235S, respectively.

The kinetic analysis of the effects of increasing concentrations of glucose and xylose over the stimulatory range (ie. 0-MC_max_) on the activity of Bglhi and mutants revealed that the stimulation occurred through non Michaelis-Menten kinetics (n_H_>1, Table 3). The values of K_aGlc_ and K_aXyl_ determined for the N89Y/H307Y and H307Y mutants were about 2.0 to 4.0-fold higher than those estimated for the wild type Bglhi. In contrast, the A141T/N235S and N235S mutants showed reduced K_aGlc_ and K_aXyl_ values as compared to Bglhi. Remarkably, the D237V/P389H/E395G/K475R mutant showed a similar K_aXyl_, as compared to Bglhi but apparently does not present a stimulatory glucose binding site, while the D237V mutant presented K_a_ values about 2.0-fold higher for both monosaccharides.

Fig 3A shows the effect of increasing concentrations of *p*NP-Glc on the activity of Bglhi and mutants in the presence of fixed concentrations of glucose or xylose corresponding to MC_max_ (except for D237V/P389H/E395G/K475R, where the glucose concentration was equal to MT), and the kinetic parameters are summarized in Table 2. The presence of either monosaccharide resulted in increased values of K_M*p*NP-Glc_ in all mutants (Table 2), indicating that even at a concentration in which their stimulatory effect is maximal, both glucose and xylose compete with *p*NP-Glc for binding to the catalytic site. Despite these common properties, the patterns of competition between the substrate and the monosaccharides were different for each mutant.

### Kinetic analysis of Bglhi and mutants using cellobiose as substrate

**Fig 4:**
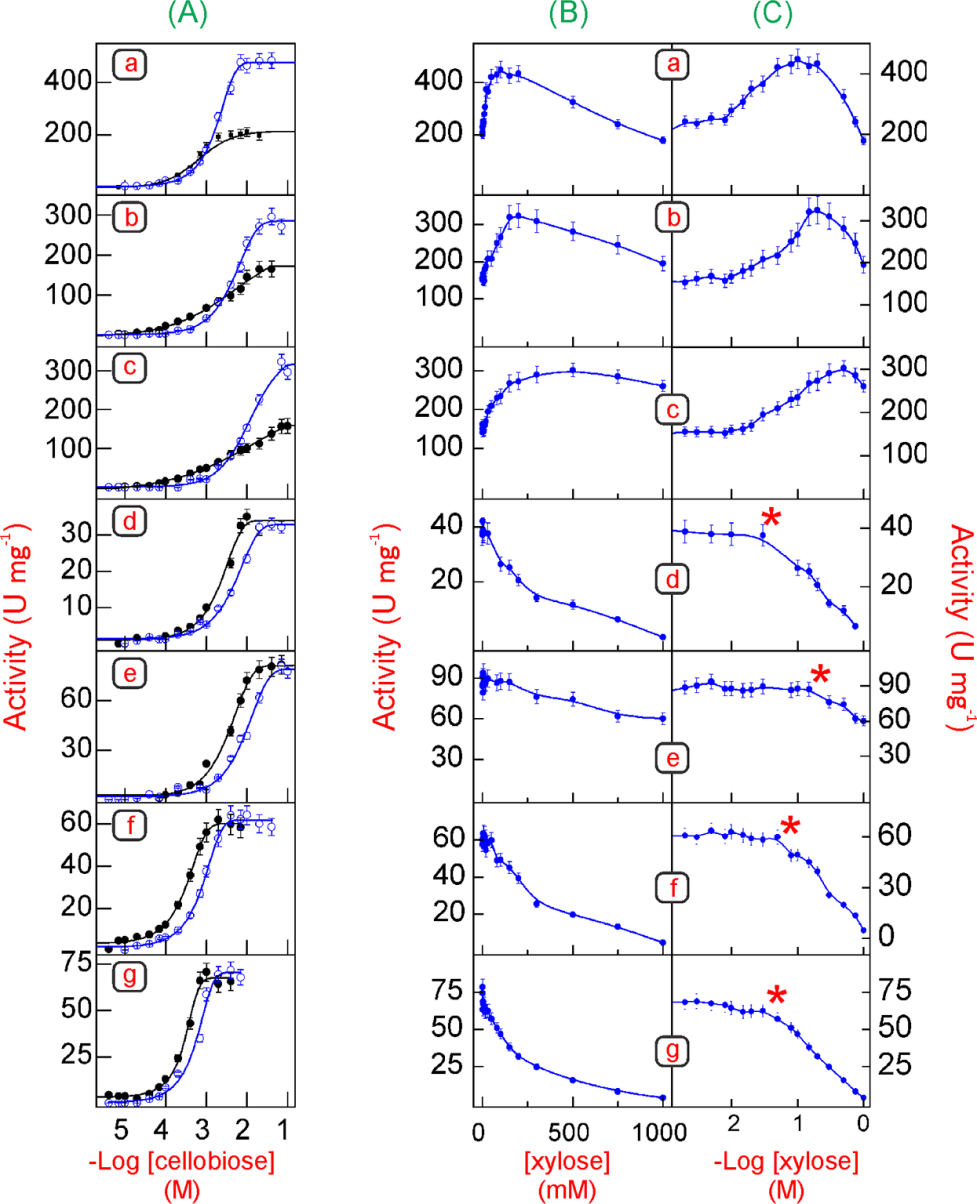
Effect of increasing concentrations of cellobiose and xylose on the cellobiase activity. (a) Bglhi, (b) N89Y/H307Y, (c) H307Y, (d) D237V/P389H/E395G/K475R, (e) D237V, (f) A141T/N235S, (g) N235S. **(A)** Effect of increasing concentrations of cellobiose on the cellobiase activity, in the absence (black lines and dots) or in the presence of fixed concentrations of xylose (blue lines and dots). The fixed concentrations of xylose were equal to MC_max_ (Bglhi, N89Y/H307Y and H307Y) or MT (D237V/P389H/E395G/K475R, D237V, A141T/N235S and N235S). **(B and C)** Effect of increasing concentrations of xylose on the cellobiase activity of each enzyme at 90–95% saturating concentrations of cellobiose (5 mM for Bglhi, 40 mM for N89Y/H307Y, 70 mM for H307Y and D237V, 10 mM for D237V/P389H/E395G/K475R, 2 mM for A141T/N235S and 1 mM for N235S). The red asterisks (*) in Fig C indicate the MT concentrations. The experiments were repeated three times using three separate pure enzyme preparations. Each point represents the mean of duplicate assays ± SD (note that the error bars are not visible, as they lie within the area of the symbol).

The cellobiase activity of Bglhi was stimulated by cellobiose through non Michaelis-Menten kinetics (Fig 4 and Table 4). Apparent positive cooperativity was observed for the stimulation of Bglhi, and the A141T/N235S and N235S mutants (n_H_>1), while an apparent negative cooperativity was seen for the stimulation of the N89Y/H307Y and H307Y mutants (Table 4), possibly reflecting the accumulation of transglycosylation products that resulted in a gradual increase of the rate of free glucose liberation as the substrate concentration increased. The V_max_ for cellobiase activity of the N89Y/H307Y and H307Y mutants was essentially unaltered as compared to the wild type Bglhi. In contrast, all other mutants presented lower V_max_ values. Furthermore, the K_Mcellobiose_ values obtained for the A141T/N235S and N235S mutants were ~1.6-fold lower as compared to the Bglhi. In contrast, the N89Y/H307Y, H307Y, D237V/P389H/E395G/K475R and D237V mutants showed higher K_Mcellobiose_ than Bglhi (Table 4).

**Table 4:** Kinetic parameters for the stimulation of the cellobiase activity of Bglhi and mutants by cellobiose in the presence or absence of fixed concentrations of xylose.

Enzyme	[Xylose]	V_max_	K_Mcellobiose_	k_cat_/K_Mcellobiose_	n_H_
	(mM)	(U mg^-1^)	(mM)	(s^-1^mM^-1^)	
Bglhi	0	213.3 ± 6.4	0.53 ± 0.02	376.29 ± 19.67	1.4
	100	499.5 ± 15.1	1.76 ± 0.05	265.36 ± 16.64	1.5
N89Y/H307Y	0	184.9 ± 11.1	2.41 ± 0.15	72.03 ± 9.38	0.6
	200	314.6 ± 18.9	5.02 ± 0.31	58.83 ± 7.63	1.1
H307Y	0	176.4 ± 12.3	5.11 ± 0.36	32.34 ± 4.84	0.6
	500	348.2 ± 24.1	12.0 ± 0.86	27.13 ± 4.07	1.1
D237V/P389H/E395G/K475R	0	37.1 ± 3.3	2.23 ± 0.21	15.77 ± 3.05	1.2
	30	37.1 ± 3.4	5.44 ± 0.49	6.42 ± 1.24	1.1
D237V	0	85.8 ± 4.3	3.01 ± 0.15	26.74 ± 2.85	1.1
	300	87.2 ± 4.4	10.12 ± 0.51	8.07 ± 0.87	1.1
A141T/N235S	0	63.1 ± 3.8	0.34 ± 0.02	173.53 ± 22.09	1.6
	50	65.4 ± 4.6	0.85 ± 0.06	71.94 ± 10.84	1.6
N235S	0	72.5 ± 3.3	0.32 ± 0.01	225.96 ± 17.39	2.0
	30	72.9 ± 2.9	0.52 ± 0.03	131.08 ± 13.66	1.5

Xylose concentrations correspond to MC_max_ (Bglhi, N89Y/H307Y and H307Y) or MT (D237V/P389H/E395G/K475R, D237V, A141T/N235S and N235S). Each experimental kinetic curve was repeated three times, using three separate preparations of the pure enzyme, where each activity assay was performed in duplicate. The kinetic parameters are given as means ± SD of the values calculated for each repetition (n = 3).

The effect of increasing xylose concentrations (0–1000 mM) on the cellobiase activity of Bglhi and mutants was also investigated (Fig 4B and 4C). Xylose stimulated the release of free glucose by Bglhi, N89Y/H307Y and H307Y by 230%, 170% and 200%, respectively, with MC_max_ values of 100, 200 and 500 mM, respectively. Above the MC_max_, a decreased specific activity was observed, and in the presence of 1000 mM xylose Bglhi exhibited 78% of the control activity, while the N89Y/H307Y and H307Y mutants maintained activities above the control (Fig 4B). Although glucose liberation by the other mutants was not increased in the presence of xylose (Fig 4B and 4C), tolerance to the monosaccharide was maintained, and the activities of the D237V/P389H/E395G/K475R, D237V, A141T/N235S and N235S mutants remained almost constant up to xylose concentrations of ~30, 300, 50 and 30 mM, respectively (MT values are indicated by the red asterisks in Fig 4C). Above the MT, however, the activities of the D237V/P389H/E395G/K475R, A141T/N235S and N235S mutants decreased rapidly with IC_50_ values of ~200, 270 and 170 mM xylose, which are significantly lower than those determined using *p*NP-Glc as substrate. The D237V mutant presented 70% of the control activity in the presence of 1000 mM xylose.

The stimulation of the cellobiase activity of Bglhi, N89Y/H307Y and H307Y by xylose within the stimulatory range (0-MC_max_) and at 90–95% saturating concentrations of cellobiose occurred through non Michaelis-Menten kinetics (n_H_>1). The values of K_aXyl_ determined for Bglhi, and the N89Y/H307Y and H307Y mutants were 19.3 ± 0.87 mM, 63.3 ± 3.2 mM and 76.7 ± 4.1 mM, respectively, which is consistent with the higher tolerance of the mutant enzymes to this monosaccharide.

The effect of increasing concentrations of cellobiose on the cellobiase activity of Bglhi and mutants in the presence of fixed concentrations of xylose corresponding to the MC_max_ (Bglhi, N89Y/H307Y and H307Y) or MT (D237V/P389H/E395G/K475R, D237V, A141TN235S and N235S) is shown in Fig 4A. Non Michaelis-Menten behavior was clearly observed for the stimulation of activity of Bglhi, and the A141T/N235S and N235S mutants by cellobiose (n_H_>1). The V_max_ values for liberation of free glucose from cellobiose in the presence of xylose for Bglhi, and the N89Y/H307Y and H307Y mutants increased by 2.3-, 1.7- and 2.0-fold, respectively, as compared to those in absence of the monosaccharide (Table 4). In contrast, V_max_ values determined in the presence or absence of xylose for the other mutants were essentially unchanged. However, as observed for *p*NP-Glc, the values of K_Mcellobiose_ for all enzymes were significantly enhanced in the presence of xylose (see Table 4), suggesting that xylose competes with the natural substrate for binding to the active site.

### Investigation of transglycosylation activity

The fractional hydrolysis (%H) and transglycosylation (%T) activities of the Bglhi and mutants at 90–95% saturating concentrations of *p*NP-Glc in the absence or presence of xylose at MC_max_ are presented in Table 5, together with the quantity of *p*NP^-^ and glucose released after 5 min reaction times. In the absence of xylose, the wild type Bglhi, and the D237V/P389H/E395G/K475R and D237V mutants predominantly followed the hydrolysis route, as demonstrated by the similar rates of *p*NP^-^ and glucose release and the low %T values. In the presence of xylose, increased %T values for these enzymes indicated a significant increase in the fraction of the glucosyl-enzyme intermediates following the transglycosylation route, resulting in higher rates of *p*NP^-^ liberation as compared to glucose. In contrast, high %T values were also determined for the N89Y/H307Y, H307Y, A141T/N235S and N235S mutants in the absence of xylose, demonstrating that these mutations have increased transglycosylation activity as compared to Bglhi. In these mutants, the presence of xylose only slightly increased the %T.

**Table 5:** Rates of *p*NP^-^ and glucose liberation and percentages of hydrolysis (%H) and transglycosylation (%T) of *p*NP-Glc by Bglhi and mutants.

Enzyme	No xylose				MC_max_			
	*p*NP^-^	Glucose	%H	%T	*p*NP^-^	Glucose	%H	%T
	(nmol)	(nmol)			(nmol)	(nmol)		
Bglhi	12.9 ± 1.1	11.1 ± 1.2	85.5 ± 7.1	14.5 ± 7.1	24.9 ± 2.1	8.1 ± 0.7	32.4 ± 2.5	67.6 ± 2.5
N89YH/307Y	12.2 ± 1.2	3.8 ± 0.2	30.9 ± 2.9	69.1 ± 2.9	31.5 ± 2.9	6.3 ± 0.6	20.0 ± 1.8	80.0 ± 1.8
H307Y	12.8 ± 1.0	4.3 ± 0.2	33.2 ± 2.7	66.8 ± 2.7	33.7 ± 2.8	7.3 ± 0.8	21.7 ± 1.9	78.3 ± 1.9
D237V/P389H/E395G/K475R	11.0 ± 0.9	10.9 ± 0.8	99.5 ± 8.5	0.5 ± 8.5	15.9 ± 1.1	10.9 ± 1.0	68.8 ± 5.4	31.2 ± 5.4
D237V	8.6 ± 0.6	8.4 ± 0.4	98.0 ± 8.9	2.0 ± 8.9	12.9 ± 1.0	5.5 ± 0.2	42.8 ± 3.7	57.2 ± 3.7
A141TN235S	8.4 ± 0.5	2.9 ± 0.1	34.7 ± 2.2	65.3 ± 2.2	11.1 ± 0.9	1.8 ± 0.1	16.1 ± 1.1	83.9 ± 1.1
N235S	10.2 ± 0.9	5.0 ± 0.1	48.8 ± 3.5	51.2 ± 3.5	14.5 ± 1.1	4.5 ± 0.2	31.0 ± 2.1	69.0 ± 2.1

The assays were repeated 6 times. *p*NP^-^ and glucose are expressed as total quantity (in nmols) released after 5 minutes. The %H and %T are given as the means ± SD of the values calculated for each assay (n = 6).

**Fig 5:**
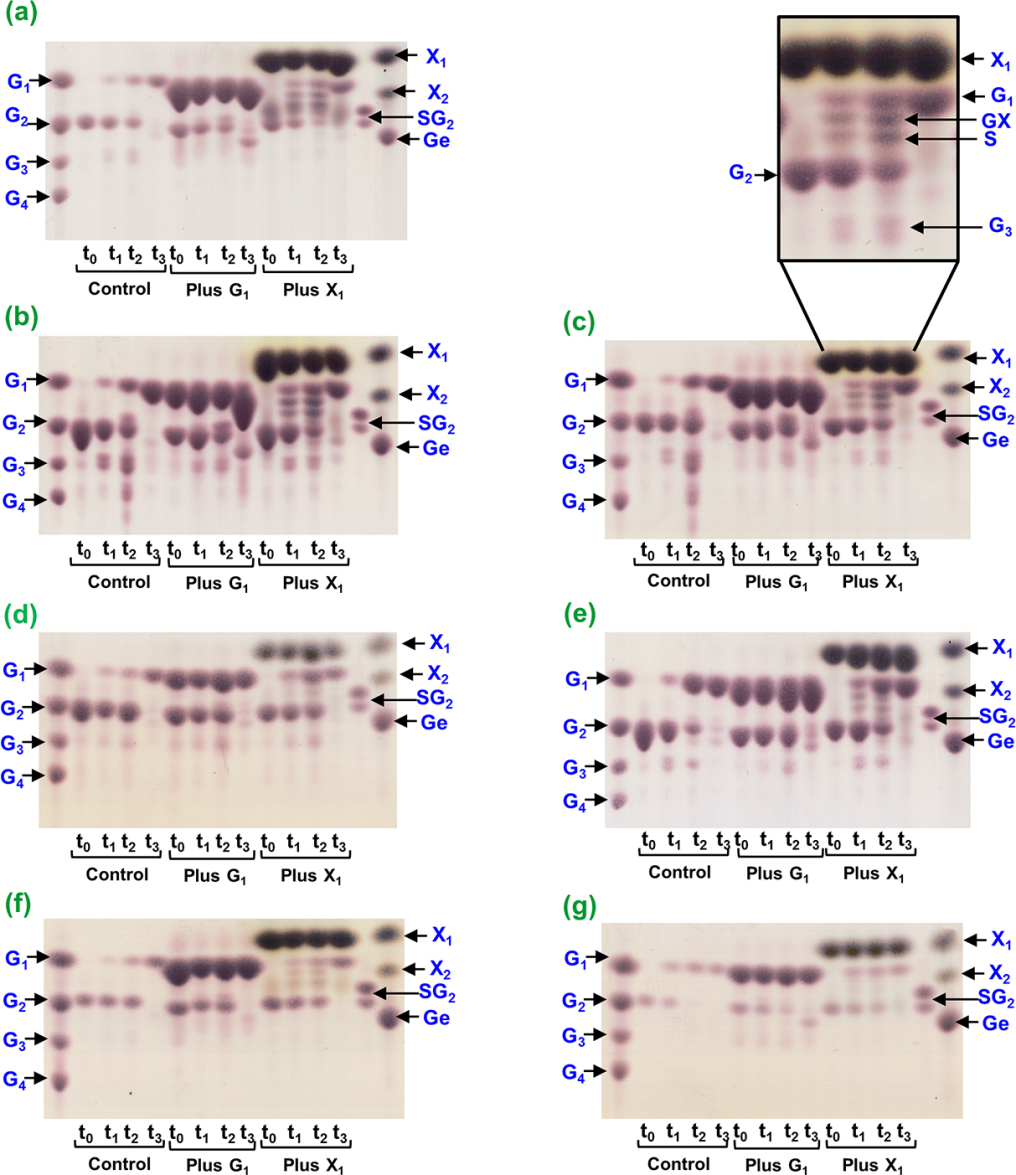
Time-course analysis of the reaction products formed by Bglhi and mutants against cellobiose. (a) Bglhi, (b) N89Y/H307Y, (c) H307Y, (d) D237V/P389H/E395G/K475R, (e) D237V, (f) A141T/N235S, (g) N235S. The reactions were performed at 90–95% saturating concentrations of cellobiose (see legend to Fig 4) in the absence (indicated as “control”) or presence of glucose (indicated as “Plus G_1_”) or xylose (indicated as “Plus X_1_”). The final concentrations of each monosaccharide were equal to MC_max_ (Bglhi, N89Y/H307Y and H307Y) or MT (D237V/P389H/E395G/K475R, D237V, A141T/N235S and N235S). The reaction times were 0 (lanes t_0_), 5 min (lanes t_1_), 10 min (lanes t_2_) and 24 h (lanes t_3_). Standards: G_1_, glucose; G_2_, cellobiose; G_3_, cellotriose; G_4_, cellotetraose; Ge, gentibiose; X_1_, xylose; X_2_, xylobiose; SG_2_, equimolar mixture of sophorose and cellobiose. The volumes of each aliquot applied to the TLC plates were adjusted aiming the best visualization of the products.

The products formed by the Bglhi and mutants with the cellobiose substrate in the absence and presence of xylose or glucose at MC_max_ (Bglhi, N89Y/H307Y and H307Y) or MT (D237V/P389H/E395G/K475R, D237V, A141T/N235S and N235S) were investigated by TLC (Fig 5). Although the wild type Bglhi predominantly formed G_1_ in the absence of either glucose or xylose, faint stains corresponding to G_3_ were visible in the TLC experiments (Fig 5A, control). In contrast, transglycosylation products were clearly observed when the reaction was performed in the presence of 100 mM xylose (Fig 5A, Plus X_1_), with the preferential formation of disaccharides. After long reaction times, glucose was the main reaction product either in the absence (Fig 5A, control, lane t_3_) or presence of xylose (Fig 5A, Plus X_1_, lane t_3_), indicating that over longer reaction times the Bglhi hydrolyzed previously formed transglycosylation products. Some transglycosylation products were also observed when glucose was added to the reaction medium (Fig 5A, Plus G_1_), however the high sample viscosity and intense glucose spot prevented their reliable separation and visualization.

In contrast to the wild type Bglhi, transglycosylation products (mostly G_3_ and G_4_) were clearly observed with the N89Y/H307Y (Fig 5B, control) and H307Y (Fig 5C, control) mutants in the absence of either glucose or xylose. In the presence of xylose, low levels of G_3_ and two other transglycosylation products (presumably disaccharides, one of them corresponding to S) were clearly observed (Fig 5B and 5C, Plus X_1_; and amplified region of Fig 5C). Thus, although the TLC pattern resembled that observed for Bglhi in the presence of xylose (Fig 5A, Plus X_1_), the stained regions were more intense. As observed with the Bglhi, G_1_ was the main product detected after long reaction times for the N89Y/H307Y and H307Y mutants, both in the absence (Fig 5B and 5C, control, line t_3_) or the presence of xylose (Fig 5B and 5C, Plus X_1_, line t_3_). Although the high sample viscosity in the presence of glucose impaired the visualization of spots located between G_1_ and G_2_ (Fig 5B and 5C, Plus G_1_), spots were observed corresponding to G_3_ after 5 and 10 min (Fig 5B and 5C, Plus G_1_, lines t_1_ and t_2_) and G_2_ after 24 h (Fig 5B and 5C, Plus G_1_, line t_3_).

In the absence of glucose or xylose, the D237V/P389H/E395G/K475R (Fig 5D, control) and D237V (Fig 5E, control) mutants predominantly followed the hydrolysis route releasing G_1_, and only low levels of G_3_ (Fig 5A, control). Furthermore, G_1_ was clearly the main product even when the reaction was performed in the presence of xylose (Fig 5B and 5E, Plus X_1_), although some weak stains corresponding to transglycosylation products (disaccharides and G_3_) were observed for both mutants under this condition. In all experiments after long incubation times, glucose was essentially the only product observed (Fig 5D and 5E, line t_3_).

**Fig 6:**
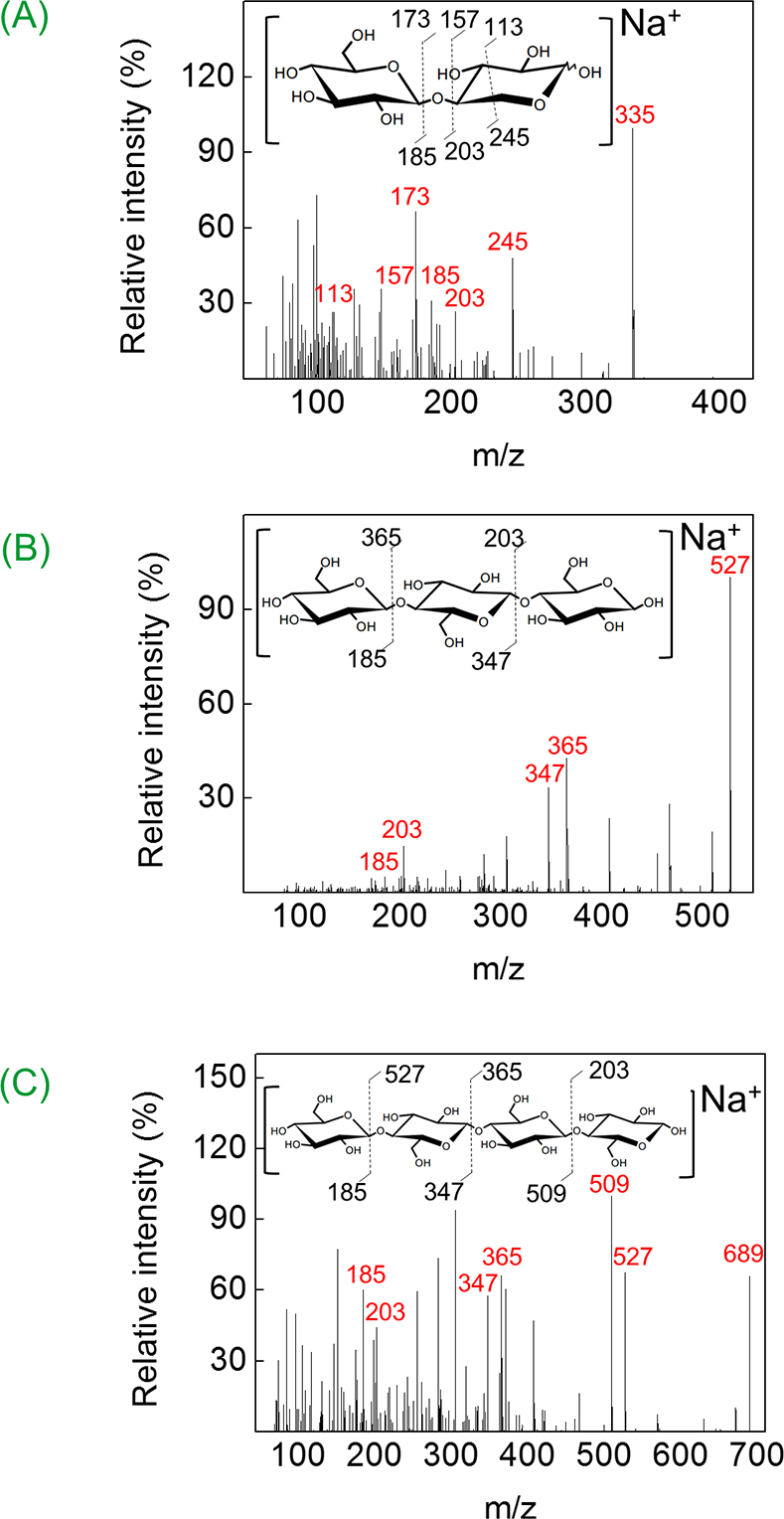
**Tandem mass spectrometry analysis of glucopyranosyl-xylose (A), cellotriose (B) and cellotetraose (C).** The sodium adducts of glucopyranosyl-xylose (*m/z* 335), cellotriose (*m/z* 527) and cellotetraose (*m/z* 689) were analyzed by MS/MS and the proposed interpretation of mass spectra are indicated on the structures.

The A141T/N235S (Fig 5F, control) and N235S (Fig 5G, control) mutants preferentially followed the hydrolysis route in the absence of xylose and glucose, with G_1_ as the only reaction product. Some disaccharide transglycosylation products were observed in the presence of xylose (Fig 5F and 5G, Plus X_1_), although at reduced levels as compared to the N89Y/H307Y and H307Y mutants. Under all experimental conditions, G_1_ was essentially the only product released by A141T/N235S (Fig 5F, lanes t_3_) and N235S (Fig 5G, lanes t_3_) mutants after prolonged reaction times. The MS/MS analysis of the reaction products of Bglhi and mutants against cellobiose, both in the absence and presence of glucose, revealed the formation of trisaccharides and tetrasaccharides constituted by units of glucose (Fig 6B and 6C). In contrast, in the presence of xylose, an ion of *m/z* 335 was detected in positive ionization mode, and MS/MS analysis indicated that this corresponded to a glucopyranosyl-xylose disaccharide (GX, Fig 6A). Based on this result and on the migration pattern of the standards used in the TLC analyses, we assigned a spot in the TLC to GX (see the amplified region of Fig 5C). No evidence was found for the formation of trisaccharides or tetrasaccharides containing xylose units by Bglhi or the mutant enzymes.

## Discussion

The crystal structure of the Bglhi (PDB 4MDO, [29]) conserves many features typical of the GH1 family β-glucosidases. The -1 glycone binding region together with the catalytic residues E166 (the catalytic acid/base) and E377 (the catalytic nucleophile) lie at the base of a deep pocket lined with polar and non-polar residues (see Fig 2). These residues optimize both specific hydrogen bonding interactions with equatorial hydroxyl groups of the glycosyl group, and aromatic-sugar stacking interactions with the pyranose ring. Immediately adjacent to the glycone site, the +1 and +2 aglycone binding sites are characterized by a broadening of the pocket to form an antechamber, and a crystal structure of the Bglhi has identified a glucose molecule that is located within this region [29].

Previous biophysical and kinetic investigations of the native Bglhi from *H*. *insolens* suggested that allosteric interactions between modulator and catalytic sites could be involved in the glucose/xylose stimulation of *p*NP-glucosidase activity [34]. The recombinant Bglhi is also stimulated by glucose/xylose [15], and a detailed kinetic analysis of the stimulation using *p*NP-Glc as substrate (S1 File) suggests a similar mechanism to that described for the native enzyme. Using the Bglhi as an example, here we report the first study of the mechanisms of glucose/xylose stimulation of the cellobiase activity of a GH1 β-glucosidase, in which kinetic analyses suggest multiple binding sites for cellobiose and xylose. Furthermore, our results suggest that xylose modulation of the enzymatic activity might involve allosteric interactions between modulator and substrate binding sites, similar to that proposed for the *p*NP-glucosidase activity [34].

A broader view of the stimulation mechanisms of the *p*NP-glucosidase and cellobiase activities of the Bglhi has been derived from transglycosylation and kinetic data. In the absence of xylose with *p*NP-Glc as substrate, the Bglhi preferentially followed the hydrolysis route (%H of ~85%) as shown by the similar release rates of *p*NP^-^ and glucose (Table 5). However, in the presence of xylose at MC_max_, the 30% decrease in the rate of free glucose release as compared to *p*NP^-^ suggests that transglycosylation was favoured. This may be the result of xylose acted as an acceptor at high concentration thereby favouring transglycosylation and enhancing the rate of formation of the glucosyl-enzyme intermediate in response to its increased consumption in the transglycosylation step. It is worthy of note that allosteric modulation of the rates of steps 1 and 2 (Fig 1) by xylose may also occur. The %T of about 15% in the absence of xylose further indicated that *p*NP-Glc (or free glucose released from it) also acted as an acceptor in transglycosylation reactions, suggesting that besides binding to the -1 glycone/+1 aglycone sites, *p*NP-Glc may also bind to the +1/+2 aglycone sites. The monosaccharides simultaneously compete with *p*NP-Glc for binding to the -1 glycone and/or the +1/+2 aglycone sites, as confirmed by the increased K_MpNP-Glc_ determined in the presence of xylose or glucose at the MC_max_ (Table 2). Above the MC_max_, the gradual decline of the *p*NP-glucosidase activity as the xylose or glucose concentration increases results from increasing competition of the monosaccharide with *p*NP-Glc for binding to the -1 glycone/+1 aglycone sites. At the MT, the stimulating effect of xylose or glucose is exactly counteracted by the reduced substrate binding to the enzyme. The lower value of K_aGlc_ as compared to K_aXyl_ (Table 3) further suggests that glucose binds to the -1 glycone and +1/+2 aglycone sites of Bglhi with higher affinity than xylose, and is consistent with the lower MC_max_ value determined for glucose as compared to xylose. Thus, the fine modulation of the *p*NP-glucosidase activity of Bglhi by glucose and/or xylose may be attributed to competition of the substrate and the modulators for the -1 glycone and the +1/+2 aglycone binding sites, and is regulated by the relative affinities of these sites for the glucose and *p*-nitrophenol moieties of the substrate, and for the free monosaccharides.

With cellobiose as substrate (Fig 5), the main reaction product in the absence of xylose was free glucose (released at steps 1 and 2, Fig 1), with low levels of G_3_ derived from transglycosylation (Fig 1, step 3) reactions in which cellobiose acted as a glucose acceptor. In contrast, transglycosylation products (mostly disaccharides) were abundant in the presence of xylose at MC_max_, indicating stimulation of the transglycosylation step. This in turn enhances the rates of the preceding glycosil-enzyme formation (Fig 1, step 1) and hydrolysis steps (Fig 1, step 2), resulting in a net increase in the rate of free glucose liberation. With cellobiose as substrate, xylose may act as both a glucose acceptor and an allosteric effector, stimulating step 1 (and eventually step 2). As proposed for *p*NP-Glc, the cellobiose may bind to either the -1/+1 sites or the +1/+2 sites of Bglhi, and xylose at concentrations up to the MC_max_ stimulates the rate of glucose release by acting as a glucose acceptor at the +1 aglycone site and/or an allosteric modulator at other aglycone sites. Simultaneously, xylose competes with cellobiose for binding to the -1 and/or the +1/+2 sites, as shown by the increased K_Mcellobiose_ determined in the presence of xylose at MC_max_ (Table 4). The gradual decrease of the activity at xylose concentrations above MC_max_ may result from increasing competition with the substrate at the -1/+1 sites, and at MT the stimulating effect of xylose is compensated by less effective cellobiose binding to the enzyme. As with the *p*NP-Glc, the fine modulation of cellobiase activity of Bglhi by xylose derives from the competition between cellobiose and xylose for binding to the -1 glycone and the +1/+2 aglycone sites, and depends on the relative affinities of these sites for the free monosaccharide and the cellobiose.

Various effects contribute to the xylose/glucose stimulation of the wild type Bglhi, including allosteric interactions, competition between the substrate and the monosaccharides for binding to different sites on the enzyme molecule, and stimulation of the transglycosylation activity. Although it has been proposed that these modulatory mechanisms may act either separately [19,20, 26,34,36, 37] or together [24] to stimulate the *p*NP-glucosidase activity of GH1 β-glucosidases, this is the first study of the stimulation mechanisms of the cellobiase activity. Results from the Bglhi mutants N235S, A141T/N235S, D237V and D237V/P389H/E395G/K475R, indicate that the glucose/xylose stimulation of *p*NP^-^ liberation from *p*NP-Glc is not always correlated with stimulation of the glucose release from cellobiose. Crucially, this demonstrates that measurement of the *p*NP-glucosidase activity is not reliable for evaluating the potential for stimulation of cellulose hydrolysis by β-glucosidases. Our results strongly suggest that an accurate description of the modulatory mechanisms of GH1 β-glucosidases depends on studies performed using the natural substrate.

All three single Bglhi mutations (H307Y, D237V and N235S) presented altered patterns of glucose or xylose stimulation and all these substitutions are located in a cluster close to the +1/+2 aglycone binding sites. These mutations directly affected the balance between the reaction rates of transglycosylation and hydrolysis in the absence of xylose, with either *p*NP-Glc or cellobiose as substrate. This indicates that the topology and surface properties of the +1/+2 aglycone binding sites are critical determinants of the reaction route followed by the wild type Bglhi. Nevertheless, in all mutant enzymes, allosteric effects, competition between the substrate and the monosaccharides for binding to different sites on the enzyme molecule, and stimulation of the transglycosylation activity all contribute to the xylose/glucose modulation of the *p*NP-glucosidase and cellobiase activities.

In the absence of xylose and with *p*NP-Glc as substrate, the N89Y/H307Y and H307Y mutants showed similar V_max_ values to the wild type Bglhi (Table 2), yet both mutants presented significantly increased rates of transglycosylation and decreased rates of hydrolysis (Table 5). These mutants also presented lower K_M*p*NP-Glc_ values (Table 2), and since the substrate can bind to either the -1 glycone/+1 aglycone sites or the +1/+2 aglycone sites, an increased affinity for the substrate at the +1/+2 aglycone sites may contribute to the reduced K_M*p*NP-Glc_, in agreement with their preference for transglycosylation. Xylose further increased transglycosylation in the N89Y/H307Y and H307Y mutants, and stimulation of the *p*NP^-^ release apparently occurred through mechanisms similar to those for the wild type Bglhi. As compared to the wild type Bglhi, the higher K_aXyl_, K_aGlc_, MC_max_ and MT values determined for the N89Y/H307Y and H307Y mutants (Table 3) support the proposal that these mutants present higher affinity for *p*NP-Glc at the +1/+2 aglycone binding sites, since higher concentrations of xylose and glucose are needed to efficiently compete with the substrate. These mutants reveal that the relative affinities of a GH1 β-glucosidase for xylose, glucose and the substrate at the -1 glycone, and the +1/+2 aglycone binding sites are crucial to determine the range of stimulatory concentrations and the tolerance to the monosaccharides.

With the cellobiose substrate, the N89Y/H307Y and H307Y mutants present significantly higher transglycosylation in the absence of xylose as compared to the wild type Bglhi (Fig 5). The rapid formation of cellooligosaccharides with 3 to 5 glucose units corroborates with an increased affinity for saccharides at the +1/+2 aglycone binding sites. Although the V_max_ for the cellobiase activity was almost unaltered, the mutants showed significantly higher K_Mcellobiose_ values as compared to the wild type Bglhi (Table 4). Apparently, the mutants present similar affinities for cellobiose at the -1 glycone/+1 aglycone sites and the +1/+2 aglycone sites, leading to an equal distribution of the substrate between these binding sites. Therefore, since cellobiose hydrolysis only occurs in molecules bound to the -1 glycone/+1 aglycone sites, an apparent negative cooperativity was observed for the stimulation of N89Y/H307Y and H307Y by cellobiose. Furthermore, the products of transglycosylation are also substrates for the mutant enzymes, and low affinity binding of these oligosaccharides might also contribute to a higher apparent K_Mcellobiose_. Xylose promoted a reduction in the levels of G_3_-G_5_ and disaccharides transglycosylation products formed by the N89Y/H307Y and H307Y mutants, and it appears that in spite of the increased affinity of the +1/+2 aglycone binding sites, oligosaccharides are displaced by xylose at MC_max_. The higher K_aXyl_, MC_max_ and MT values determined for the N89Y/H307Y and H307Y mutants (Table 3) as compared to the wild type Bglhi further supports the suggestion of higher affinity of the mutants for cellobiose at the +1/+2 aglycone sites. The formation of GX unequivocally demonstrates that xylose stimulated transglycosylation activity by binding to the +1 aglycone site, but the increased rate of glucose release in the presence of xylose at MC_max_ indicates that xylose also stimulates steps 1 and/or 2 (Fig 1) of the reaction. The rapid formation of sophorose (or other glucose dimers) in the presence of xylose at MC_max_ further suggests that the +1 aglycone binding site of the mutant enzymes has a significantly higher affinity for glucose, as compared to xylose. Importantly, this is the first report of the formation of GX by a GH-1 β-glucosidase in a transglycosylation reaction.

In the absence of xylose, the D237V/P389H/E395G/K475R and D237V mutants exclusively followed the hydrolysis route with the *p*NP-Glc substrate (Table 5). The higher V_max_ for the *p*NP-glucosidase activity of these mutants, as compared to the wild type Bglhi (Table 2), may reflect a stabilization of the transition state of the first step of the catalytic cycle (Fig 1, step 1) probably due to stronger interaction of the *p*NP^-^ moiety at the +1 aglycone site. Xylose at the MC_max_ substantially increased the %T values for the D237V/P389H/E395G/K475R and D237V mutants, and the stimulation of transglycosylation apparently occurred through mechanisms similar to those proposed for the wild type Bglhi, resulting in increased *p*NP-glucosidase activities. The higher values of K_aXyl_, K_aGlc_ and MC_max_ determined for the D237V mutant (Table 3), as compared to wild type Bglhi, are consistent with a reduced binding affinity of xylose and glucose to the +1 aglycone site, while the lower MT values observed for both monosaccharides may reflect an increased affinity at the -1 glycone site. The lower K_aGlc_, as compared to K_aXyl_, and the much higher K_MpNP-Glc_ determined in the presence of glucose at MC_max_ also reflect a higher affinity for glucose binding at the -1 glycone sites of D237V as compared to xylose. Taken together, these data suggest that the D237V mutation reduced affinity of the +1 aglycone sites more for glucose than for xylose. This effect was even more apparent in the D237V/P389H/E395G/K475R mutant, where the *p*NP-glucosidase activity was not stimulated by glucose but increased 1.4-fold with xylose. Nevertheless, glucose efficiently competed with the substrate for binding to the -1 glycone site of the D237V/P389H/E395G/K475R mutant, as shown by the increased K_MpNP-Glc_ value in the presence of glucose at MC_max_ (Table 2), and strong glucose inhibition of enzyme activity at concentrations above 50 mM. It is noteworthy that a previous site directed mutagenesis study of two metagenome derived β-glucosidases have shown that residues such as aspartic acid with hydrogen bonding potential at the position homologous to 237 in Bglhi retain glucose stimulated activity [37]. In contrast, residues with side-chains having no hydrogen bonding potential, such as valine, lose their glucose stimulation [37]. Taken together, the results from the D237V/P389H/E395G/K475R and D237V mutants further corroborate the hypothesis that the affinities of the GH1 β-glucosidases for xylose, glucose or the substrate at the glycone and aglycone binding sites are important determinants of monosaccharide tolerance.

Hydrolysis of the cellobiose substrate was strongly favoured in the D237V/P389H/E395G/K475R and D237V mutants in the absence of xylose, which may be attributed to a decreased affinity for the non-reducing glucose residue of cellobiose at the +1 aglycone site of the mutants. In striking contrast with *p*NP-Glc as substrate, the V_max_ values for the cellobiase activity of both mutants were much lower than for the wild type Bglhi (Table 4), possibly due to an increased activation energy of step 1 (Fig 1). Both mutants also showed significantly increased K_Mcellobiose_, as compared to Bglhi, possibly reflecting a lower cellobiose affinity at the +1 aglycone site. The cellobiase activity of the D237V/P389H/E395G/K475R mutant was not stimulated by xylose, and the low levels of transglycosylation products formed are possibly the result of a combination of low affinity for xylose at the +1 aglycone site associated with a higher affinity for xylose at the -1 glycone site, which is also in accord with the very low MT value. In contrast, although the cellobiase activity of the D237V mutant was also not stimulated by xylose, some transglycosylation products (mainly disaccharides) were formed at low levels in the presence of xylose at the MT (Fig 5). As observed for the D237V/P389H/E395G/K475R mutant, xylose strongly competed with the substrate, as revealed by the much higher K_Mcellobiose_ determined in the presence of xylose at MT (Table 4). However, in the D237V mutant the monosaccharide apparently binds with considerably higher affinity at the +1 aglycone site, as compared to D237V/P389H/E395G/K475R, and with lower affinity at the -1 glycone sites, as revealed by the 10-fold higher MT.

The A141T/N235S and N235S mutants showed 50–65% transglycosylation with the *p*NP-Glc substrate (Table 5) in the absence of xylose, indicating that the N235S reduced the preference for the hydrolysis seen in the wild type Bglhi, and indicate a higher *p*NP-Glc affinity at the +1/+2 sites. The reduced K_M*p*NP-Glc_ observed in the A141T/N235S and N235S mutants (Table 2) may also reflect an increased affinity for the substrate at the -1 glycone site. Similar V_max_ values were observed for the *p*NP-glucosidase activities of these mutants and the wild type Bglhi, indicating that the rate of *p*NP^-^ release was not altered. This is in contrast with the increased rate of transglycosylation and decreased rate of glucose release. In the presence of xylose, the %T determined for both mutants were slightly increased and the *p*NP^-^ release was apparently stimulated by a mechanism similar to that proposed for the wild type enzyme. The reduced K_aXyl_, K_aGlc_, MC_max_ and MT values determined for the A141T/N235S and N235S mutants (Table 3) as compared to Bglhi are consistent with the binding of the monosaccharides at the -1 glycone and +1/+2 aglycone sites with higher affinities, as confirmed by the increased K_M*p*NP-Glc_ determined in the presence of xylose or glucose at MC_max_ (Table 2).

Transglycosylation products at appreciable levels were not formed with cellobiose as substrate (Fig 5) by the A141T/N235S and N235S mutants in the absence of xylose, suggesting that the N235S mutation does not alter the affinity of the +1/+2 aglycone binding sites for the natural substrate. The lower K_Mcellobiose_ determined for the mutants, as compared to Bglhi, are consistent with higher affinities for the substrate at the -1 glycone/+1 aglycone sites. The cellobiase activity of the A141T/N235S and N235S mutants was not stimulated by xylose, and only low levels of transglycosylation products were formed in the presence of xylose at MT, which is consistent with reduced affinity for the monosaccharide at the +1 aglycone site of the mutants. The lower MT values reflect higher apparent affinities for xylose at the -1 glycone site, as compared to the wild type Bglhi.

## Conclusions

Using the Bglhi in a combined protein engineering, kinetic and transglycosylation study, we have investigated the mechanisms of glucose/xylose stimulation of the cellobiase activity of a GH1 β-glucosidase, which has yielded novel insights as to the stimulation of *p*NP-glucosidase activity. Our results suggest that allosteric interactions, increased transglycosylation and competition of the monosaccharides glucose and xylose with the substrate at the -1 glycone and the +1/+2 aglycone binding sites all play a role in the stimulation mechanism. A directed protein evolution strategy has created a series of Bglhi mutants with altered catalytic properties with respect to glucose/xylose stimulation and/or tolerance. Our data suggest that the fine modulation of the activity of the Bglhi and mutants by glucose and/or xylose is regulated by the relative affinities of the glycone and aglycone binding sites for the substrate and the free monosaccharides. It is proposed that changes in the topology and physicochemical properties of the +1/+2 aglycone sites of the mutants underly the altered kinetic and transglycosylation activities of the enzyme.

## Supporting information

S1 Fig Analysis of purified Bglhi and mutants by SDS-PAGE. (DOC)

S2 Fig **Effect of temperature (A) and pH (B) on *p*NP-glucosidase activity of Bglhi and mutants.** (a) Bglhi, (b) N89Y/H307Y, (c) H307Y, (d) D237V/P389H/E395G/K475R, (e) D237V, (f) A141T/N235S, (g) N235S. The optimum catalytic temperatures were estimated in 50 mM Bis-Tris buffer, pH 6.0, containing 2 mM *p*NP-Glc. The optimum catalytic pH were estimated in McIlvaine buffer at the optimum temperatures determined for each enzyme, using 2 mM *p*NP-Glc as substrate. The values shown represent means ± SD from triplicate assays (n = 3) carried out with three separate preparations of pure recombinant enzymes (error bars are not evident, as lie within the area of the symbol).
(DOC)

S1 File Detailed kinetic analysis of the stimulation of Bglhi using *p*NP-Glc as substrate. (PDF)
