# Supplementary material for: Engineering the GH1 β-glucosidase from Humicola insolens: Insights on the stimulation of activity by glucose and xylose
Source: PLoS One. 2017 Nov 16;12(11):e0188254. doi: 10.1371/journal.pone.0188254 (PMC5690678; doi:10.1371/journal.pone.0188254)
Supplement: S1 Fig — (DOC) [file pone.0188254.s001.doc]

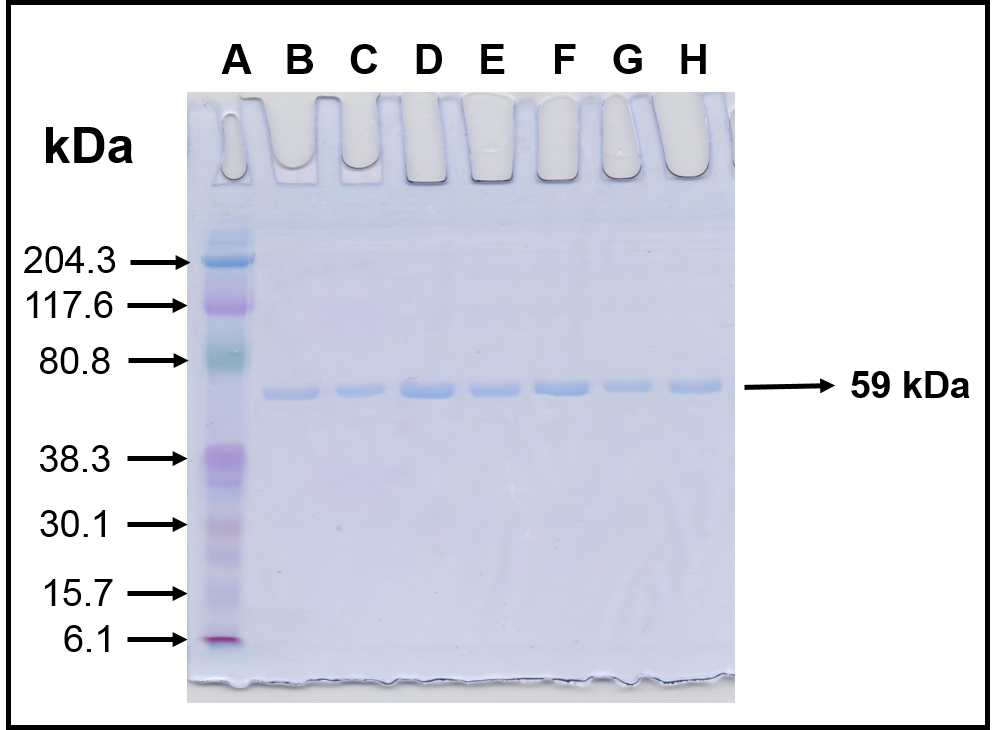


**S1 Fig. Analysis of purified Bglhi and mutants by SDS-PAGE.**

Lane A, protein molecular mass markers; lane B, Bglhi (10 µg); lane C, N89Y/H307Y (12 µg); lane D, H307Y (15 µg); lane E, D237V/P389H/E395G/K475R (15 µg); lane F, D237V (15 µg), lane G, A141T/N235S (12 µg), lane H, N235S (14 µg). Protein bands were revealed with Coomassie Brilliant Blue R.
