# Supplementary material for: Engineering the GH1 β-glucosidase from Humicola insolens: Insights on the stimulation of activity by glucose and xylose
Source: PLoS One. 2017 Nov 16;12(11):e0188254. doi: 10.1371/journal.pone.0188254 (PMC5690678; doi:10.1371/journal.pone.0188254)
Supplement: S2 Fig — Effect of temperature (A) and pH (B) on pNP-glucosidase activity of Bglhi and mutants. (a) Bglhi, (b) N89Y/H307Y, (c) H307Y, (d) D237V/P389H/E395G/K475R, (e) D237V, (f) A141T/N235S, (g) N235S. The optimum catalytic temperatures were estimated in 50 mM Bis-Tris buffer, pH 6.0, containing 2 mM pNP-Glc. The optimum catalytic pH were estimated in McIlvaine buffer at the optimum temperatures determined for each enzyme, using 2 mM pNP-Glc as substrate. The values shown represent means ± SD from triplicate assays (n = 3) carried out with three separate preparations of pure recombinant enzymes (error bars are not evident, as lie within the area of the symbol). (DOC) [file pone.0188254.s002.doc]

**
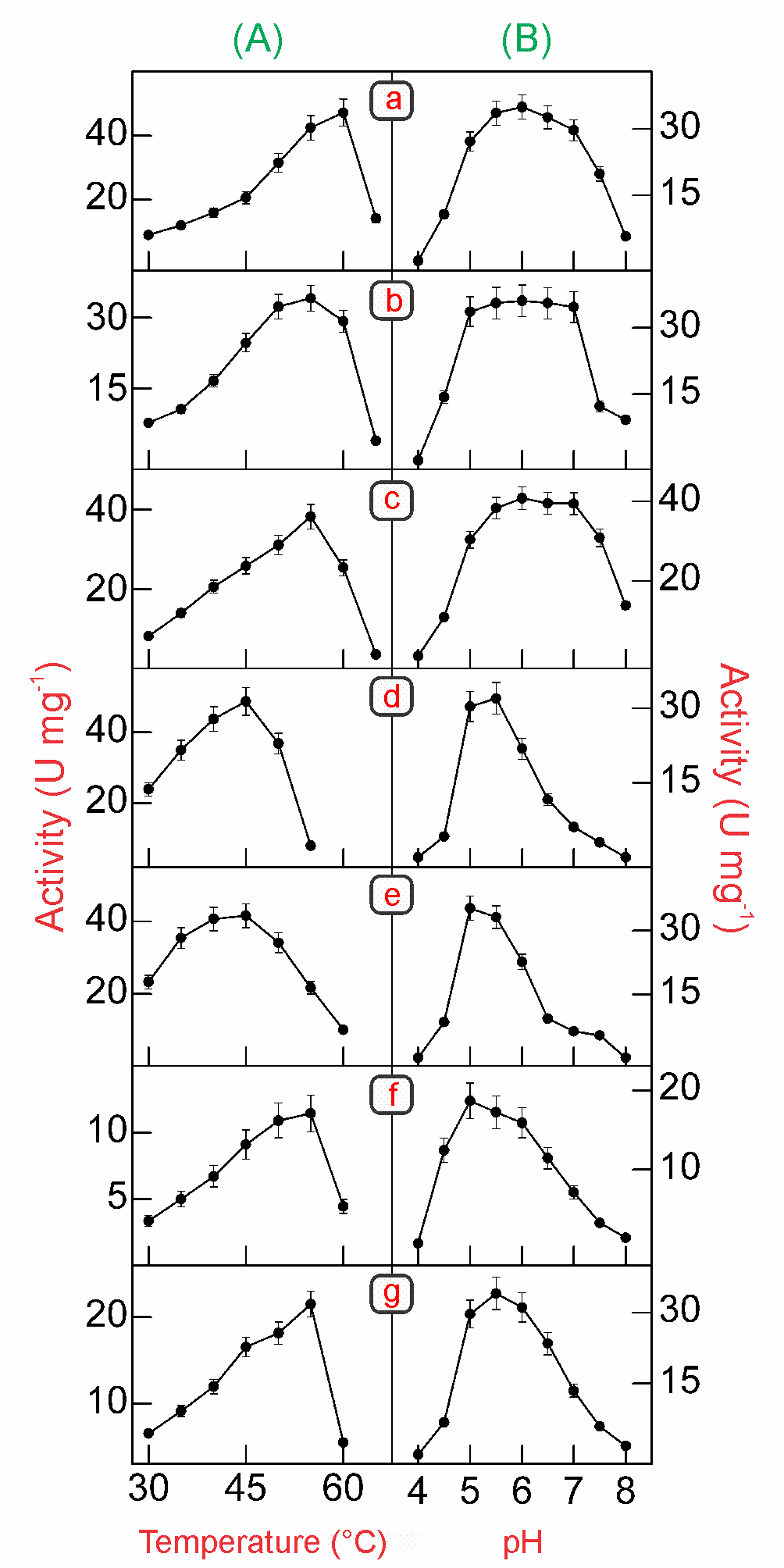
**

**S2 Fig. Effect of temperature (A) and pH (B) on *p*NP-glucosidase activity of Bglhi and mutants.** (a) Bglhi, (b) N89Y/H307Y, (c) H307Y, (d) D237V/P389H/E395G/K475R, (e) D237V, (f) A141T/N235S, (g) N235S. The optimum catalytic temperatures were estimated in 50 mM Bis-Tris buffer, pH 6.0, containing 2 mM *p*NP-Glc. The optimum catalytic pH were estimated in McIlvaine buffer at the optimum temperatures determined for each enzyme, using 2 mM *p*NP-Glc as substrate. The values shown represent means ± SD from triplicate assays (n= 3) carried out with three separate preparations of pure recombinant enzymes (error bars are not evident, as lie within the area of the symbol).
