# Supplementary material for: Engineering the GH1 β-glucosidase from Humicola insolens: Insights on the stimulation of activity by glucose and xylose
Source: PLoS One. 2017 Nov 16;12(11):e0188254. doi: 10.1371/journal.pone.0188254 (PMC5690678; doi:10.1371/journal.pone.0188254)
Supplement: S1 File — (PDF) [file pone.0188254.s003.pdf]

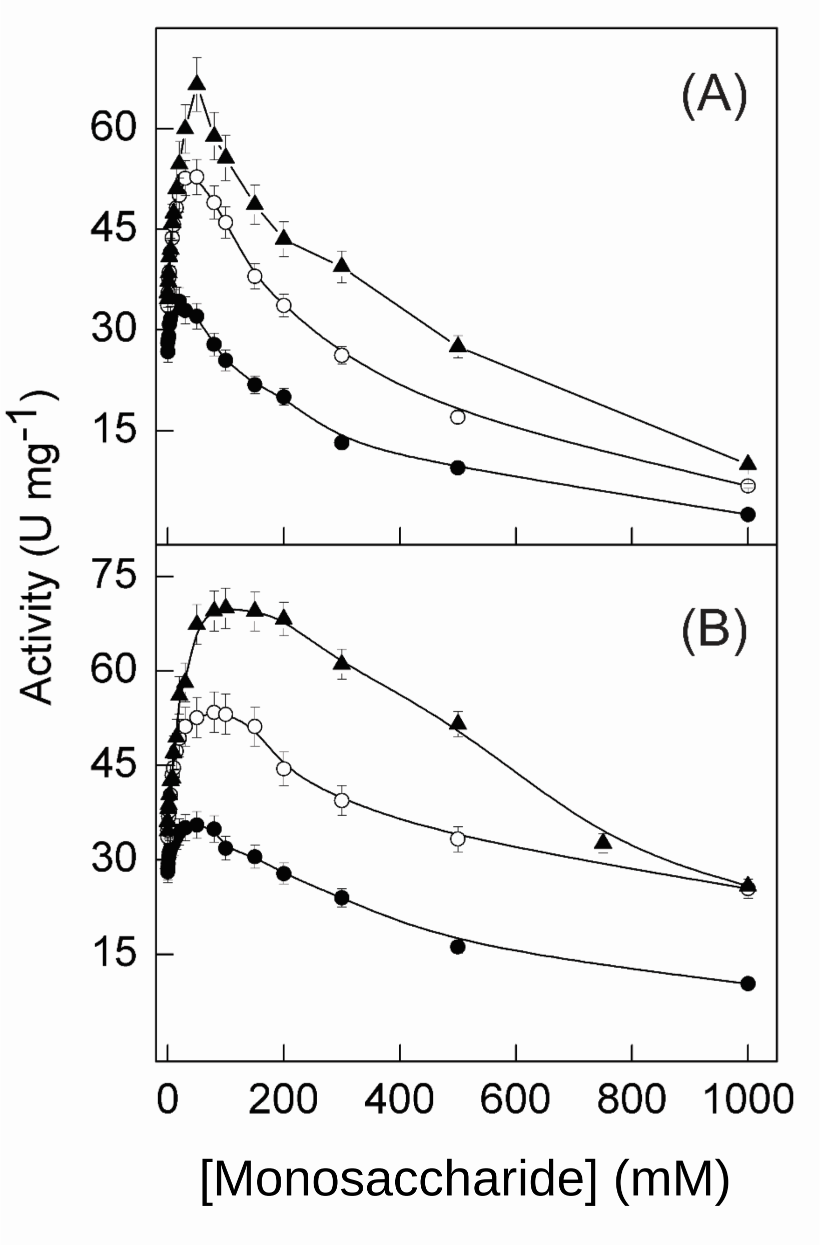

S1 Fig. Effect of increasing glucose (A) and xylose (B) concentrations on Bglhi activity at fixed *p*NP-Glc concentrations

The *p*NP-glucosidase activity was assayed at 50 °C in 50 mM Bis-Tris buffer, pH 6.0. Fixed concentrations of *p*NP-Glc were: (●) 0.5 mM, (○) 1 mM and (▲) 2 mM. The experiments were repeated three times using three separate pure enzyme preparations. Each point represents the mean of duplicate assays ± SD (error bars are not evident, as lie within the area of the symbol).

S1 Table: Kinetic parameters for the stimulation of the *p*NP-glucosidase activity of Bglhi by *p*NP-Glc in the absence or presence of fixed concentrations of glucose or xylose.

	[Monosaccharide] (mM)	V_{max} (U mg^{-1})	$K_{pNP\text{-}Glc}$ (mM)	$k_{cat}/K_{pNP\text{-}Glc}$ (s^{-1}mM^{-1})	n_H
**Control**	0	36.4 ± 1.4	0.22 ± 0.01	154.7 ± 16.1	1.2
**Glucose**	5	46.8 ± 1.9	0.25 ± 0.01	175.0 ± 15.1	1.2
	10	49.4 ± 2.4	0.28 ± 0.02	165.0 ± 21.2	1.4
	20	56.1 ± 1.7	0.36 ± 0.01	145.7 ± 9.1	1.3
	50	70.4 ± 2.8	0.50 ± 0.01	131.7 ± 8.4	1.4
**Xylose**	10	47.9 ± 1.5	0.25 ± 0.01	179.1 ± 13.7	1.3
	30	59.3 ± 2.5	0.34 ± 0.02	163.1 ± 17.6	1.5
	50	64.1 ± 1.9	0.39 ± 0.02	153.7 ± 12.0	1.4
	100	74.5 ± 3.1	0.52 ± 0.01	134.0 ± 8.7	1.4

The *p*NP-glucosidase activity was assayed at 50 °C in 50 mM Bis-Tris buffer, pH 6.0, containing glucose or xylose at the concentrations indicated. Each experimental kinetic curve was repeated three times, using three separate preparations of the pure enzyme. Each activity assay was performed in duplicate. The kinetic parameters are given as means ± SD of the values calculated for each repetition (n= 3).

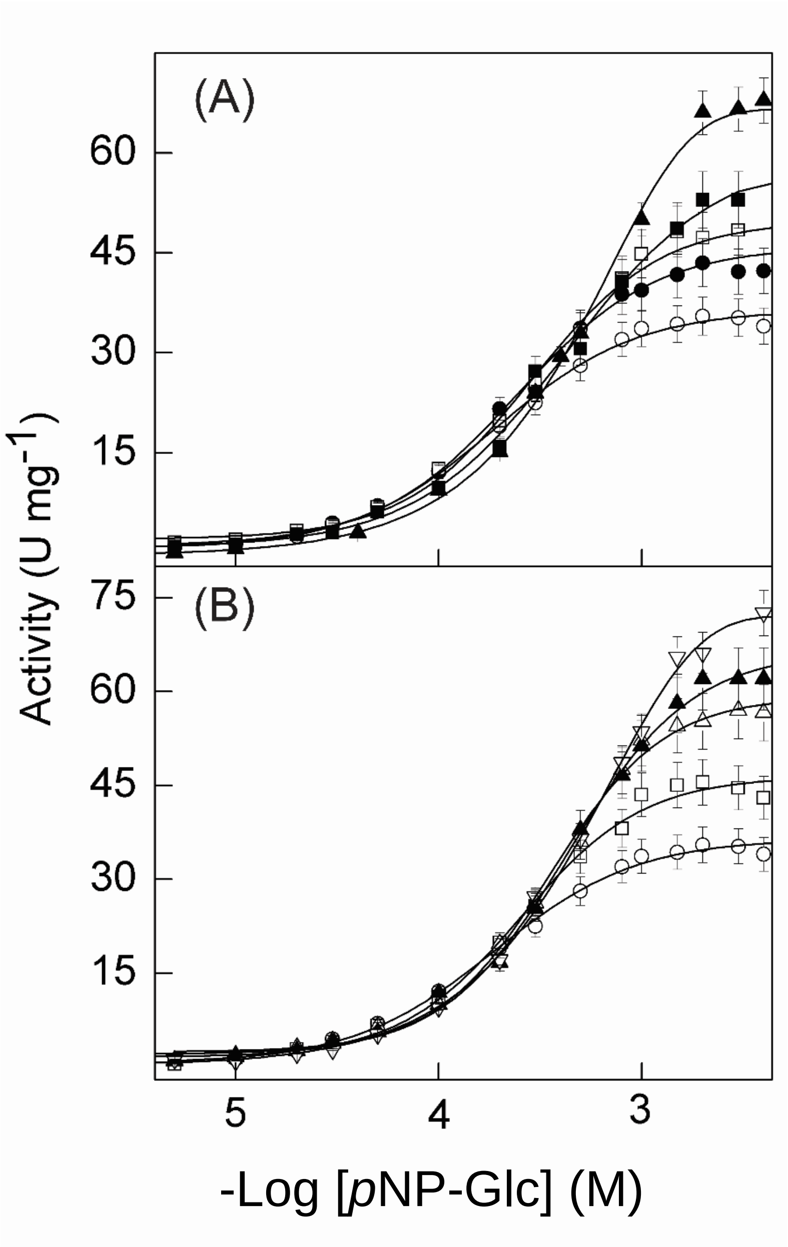

S2 Fig. Stimulation of the *p*NP-glucosidase activity of Bglhi by *p*NP-Glc in the absence or presence of fixed concentrations of glucose (A) or xylose (B)

The *p*NP-glucosidase activity was assayed at 50 °C in 50 mM Bis-Tris buffer, pH 6.0. Fixed concentrations of glucose were: (○) none, (●) 5 mM, (□) 10 mM, (■) 20 mM, (▲) 50 mM. Fixed concentrations of xylose were: (○) none, (□) 10 mM, (△) 30 mM, (▲) 50 mM, (▽) 100 mM. The experiments were repeated three times using three separate pure enzyme preparations. Each point represents the mean of duplicate assays ± SD (error bars are not evident, as lie within the area of the symbol).

S2 Table: Kinetic parameters for the stimulation of Bglhi activity by glucose and xylose in the presence of fixed concentrations of *p*NP-Glc.

Monosaccharide	[*p*NP-Glc] (mM)	V_{max} (U mg^{-1})	K_{Glc} or K_{Xyl} (mM)	n_H
**Glucose (1.0 - 100.0 mM)**	0.5	35.0 ± 1.2	4.6 ± 0.2	1.7
	1.0	56.0 ± 1.9	8.6 ± 0.4	1.2
	2.0	68.5 ± 1.6	11.4 ± 0.6	1.3
**Xylose (1.0 - 200.0 mM)**	0.5	36.6 ± 1.2	6.9 ± 0.2	1.1
	1.0	55.0 ± 2.4	9.2 ± 0.3	1.2
	2.0	74.1 ± 3.4	17.5 ± 0.8	1.2

The *p*NP-glucosidase activity was assayed at 50 °C in 50 mM Bis-Tris buffer, pH 6.0, containing *p*NP-Glc at the concentrations indicated. Each experimental kinetic curve was repeated three times, using three separate preparations of the pure enzyme. Each activity assay was performed in duplicate. The kinetic parameters are given as means ± SD of the values calculated for each repetition (n= 3).

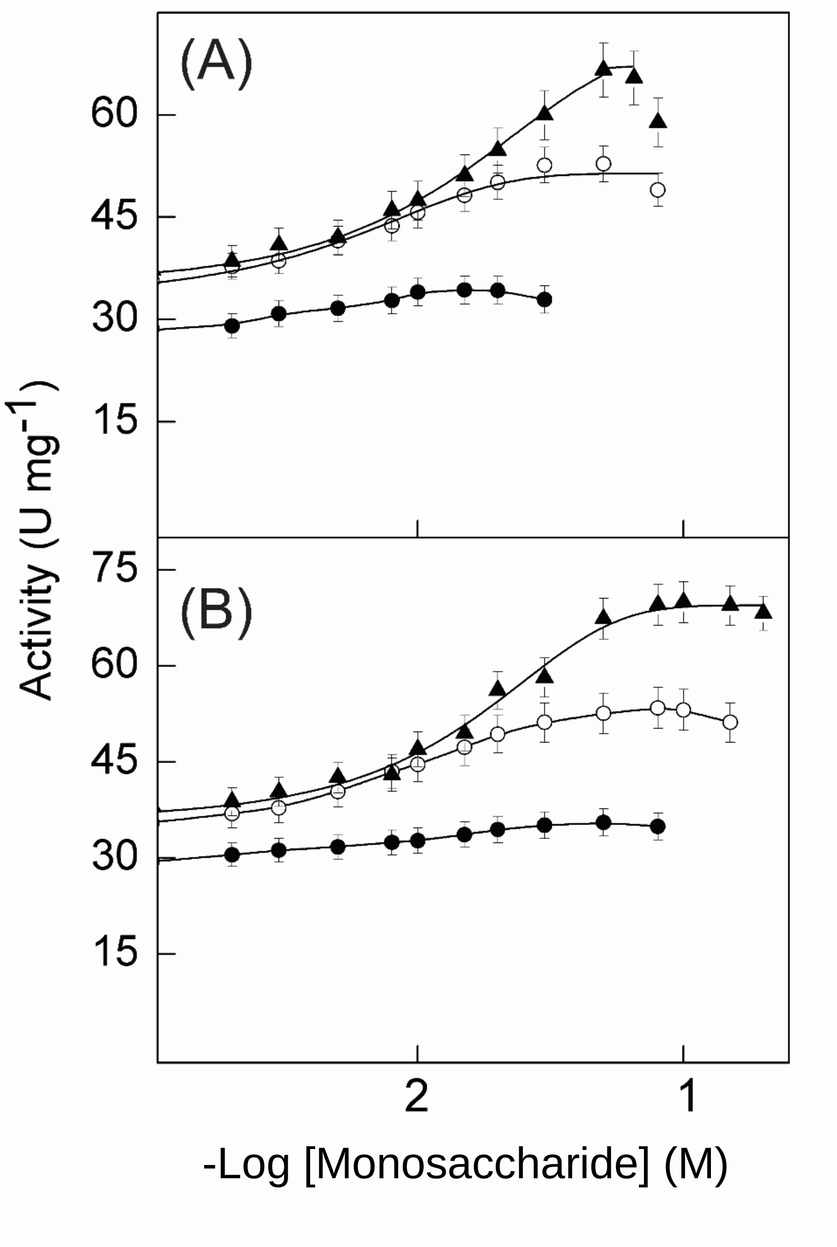

S3 Fig. Stimulation of the *p*NP-glucosidase activity of Bglhi by glucose (A) and xylose (B) at fixed *p*NP-Glc concentrations

The *p*NP-glucosidase activity was assayed at 50 °C in 50 mM Bis-Tris buffer, pH 6.0. Fixed concentrations of *p*NP-Glc were: (●) 0.5 mM, (○) 1 mM and (▲) 2 mM. The experiments were repeated three times using three separate pure enzyme preparations. Each point represents the mean of duplicate assays ± SD (error bars are not evident, as lie within the area of the symbol).

S3 Table: Kinetic parameters for the modulation of Bglhi activity by glucose and/or xylose.

[Glucose] (mM)	[Xylose] (mM)	V_{max} (U mg^{-1})	K_{Glc} or K_{Xyl} (mM)	n_H
Variable	0	68.5 ± 1.6	11.4 ± 0.6	1.3
Variable	20	65.7 ± 3.3	14.1 ± 0.4	1.5
0	Variable	74.1 ± 2.4	17.5 ± 0.7	1.2
20	Variable	72.0 ± 2.4	24.2 ± 0.7	1.4

The *p*NP-glucosidase activity was assayed at 50 °C in 50 mM Bis-Tris buffer, pH 6.0, containing 2 mM *p*NP-Glc Each experimental kinetic curve was repeated three times, using three separate preparations of the pure enzyme. Each activity assay was performed in duplicate. The kinetic parameters are given as means ± SD of the values calculated for each repetition (n= 3).

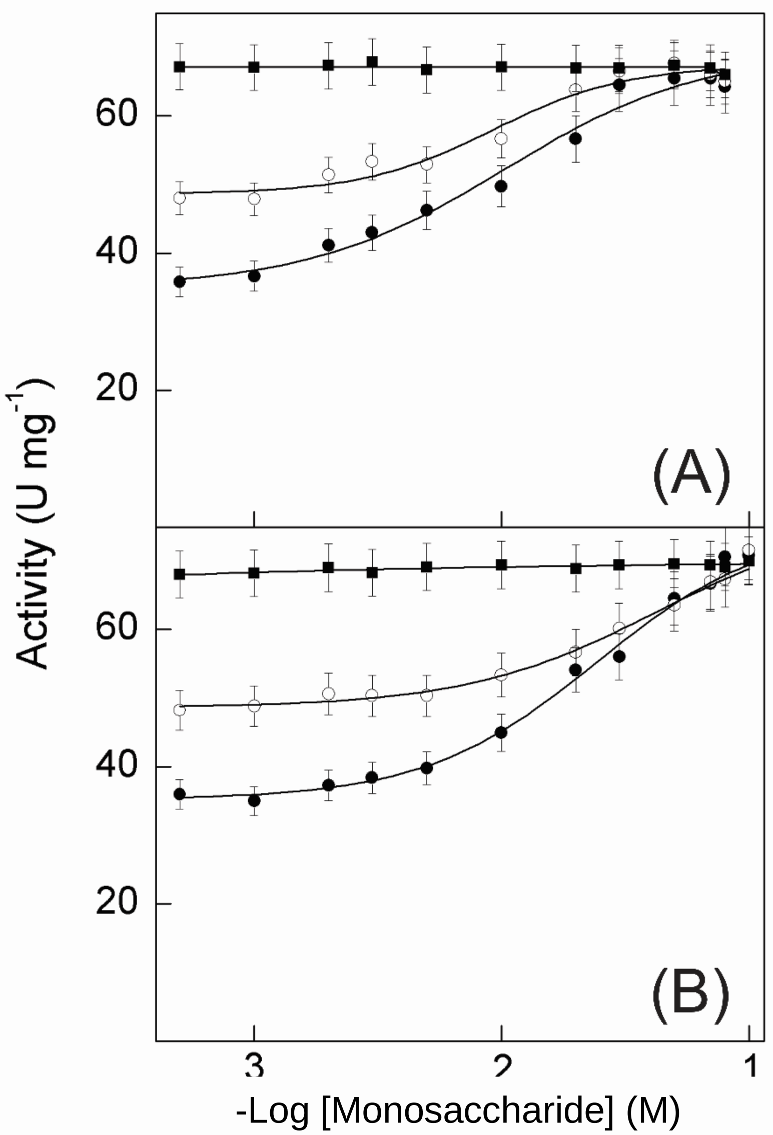

S4 Fig. Modulation of Bglhi activity by glucose (A) and xylose (B) in presence of fixed concentrations of each monosaccharide

The *p*NP-glucosidase activity was assayed at 50 °C in 50 mM Bis-Tris buffer, pH 6.0, containing 2 mM *p*NP-Glc. Fixed concentrations of (A) xylose or (B) glucose: (●) none, (○) 20 mM and (■) 50 mM. The experiments were repeated three times using three separate pure enzyme preparations. Each point represents the mean of duplicate assays ± SD (error bars are not evident, as lie within the area of the symbol).
